# Dissecting humoral immune responses to an MVA-vectored MERS-CoV vaccine in humans using a systems serology approach

**DOI:** 10.1016/j.isci.2024.110470

**Published:** 2024-07-08

**Authors:** Leonie M. Weskamm, Paulina Tarnow, Charlotte Harms, Melanie Huchon, Matthijs P. Raadsen, Monika Friedrich, Laura Rübenacker, Cordula Grüttner, Mariana G. Garcia, the MVA-MERS-S-CEF study group, Till Koch, Stephan Becker, Gerd Sutter, Edouard Lhomme, Bart L. Haagmans, Anahita Fathi, Sandra M. Blois, Christine Dahlke, Laura Richert, Marylyn M. Addo

**Affiliations:** 1 Institute for Infection Research and Vaccine Development (IIRVD), Center for Internal Medicine, University Medical Center Hamburg-Eppendorf, Hamburg, Germany; 2 Department for Clinical Immunology of Infectious Diseases, Bernhard Nocht Institute for Tropical Medicine, Hamburg, Germany; 3 German Center for Infection Research, Partner Site Hamburg-Lübeck-Borstel-Riems, Hamburg, Germany; 4 Department of Obstetrics and Fetal Medicine, University Medical Center Hamburg-Eppendorf, Hamburg, Germany; 5 Glyco-HAM, a Cooperation of Universität Hamburg, Technology Platform Mass Spectrometry and University Medical Center Hamburg-Eppendorf, Hamburg, Germany; 6 University of Bordeaux, INSERM, INRIA, BPH, U1219, Sistm, Bordeaux, France; 7 Vaccine Research Institute, Creteil, France; 8 Department of Viroscience, Erasmus University Medical Center, Rotterdam, the Netherlands; 9 Antibiotic Stewardship Team, Pharmacy of the University Medical Center Hamburg-Eppendorf, Hamburg, Germany; 10 Institute of Virology, Philipps University Marburg, Marburg, Germany; 11 German Center for Infection Research, Partner Site Gießen-Marburg-Langen, Marburg, Germany; 12 Division of Virology, Department of Veterinary Sciences, Ludwig Maximilian University Munich, Munich, Germany; 13 German Center for Infection Research, Partner Site München, Munich, Germany; 14 CHU de Bordeaux, Service d’Information Médicale, Bordeaux, France; 15 Division of Infectious Diseases, 1st Department of Medicine, University Medical Center Hamburg-Eppendorf, Hamburg, Germany

**Keywords:** Cell biology, Immune response, Immunology, Virology

## Abstract

Besides neutralizing antibodies, which are considered an important measure for vaccine immunogenicity, Fc-mediated antibody functions can contribute to antibody-mediated protection. They are strongly influenced by structural antibody properties such as subclass and Fc glycan composition. We here applied a systems serology approach to dissect humoral immune responses induced by MVA-MERS-S, an MVA-vectored vaccine against the Middle East respiratory syndrome coronavirus (MERS-CoV). Building on preceding studies reporting the safety and immunogenicity of MVA-MERS-S, our study highlights the potential of a late boost, administered one year after prime, to enhance both neutralizing and Fc-mediated antibody functionality compared to the primary vaccination series. Distinct characteristics were observed for antibodies specific to the MERS-CoV spike protein S1 and S2 subunits, regarding subclass and glycan compositions as well as Fc functionality. These findings highlight the benefit of a late homologous booster vaccination with MVA-MERS-S and may be of interest for the design of future coronavirus vaccines.

## Highlights

• MVA-MERS-S vaccination induces antibodies with neutralizing and Fc functionality
• A late booster vaccination enhances both titers and functionality of antibodies
• S1- and S2-specific IgG differ in Fc functionality and N-linked glycan composition

## Graphical abstract

**Figure:**
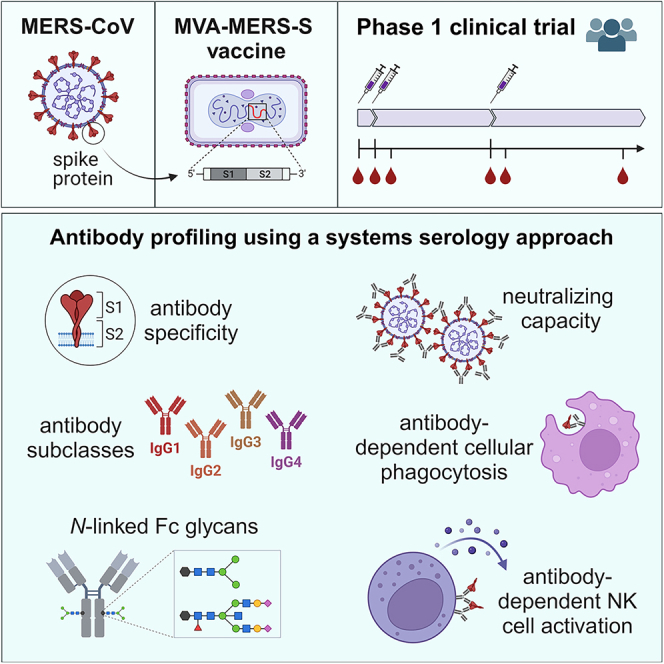

## Introduction

The Middle East respiratory syndrome coronavirus (MERS-CoV) was first isolated from a patient in Saudi Arabia in 2012, and dromedary camels have been identified as the main source of virus transmission to humans.^1^^,^^2^^,^^3^ As of October 2023, a total of 2605 laboratory-confirmed cases of human MERS-CoV infection have been reported, most of them in the Middle East.^4^ Manifestations of MERS range from unspecific, influenza-like symptoms to severe pneumonia and multiple organ failure, and are linked to a case-fatality ratio of up to 36%.^4^^,^^5^ Currently, no licensed vaccines or specific therapeutics are available to prevent or treat MERS-CoV infection and disease, even though MERS and MERS-like coronaviruses have been recently assessed as a potential pandemic threat.^5^^,^^6^^,^^7^ The MERS-CoV spike (S) protein is considered an important target for vaccine development, due to its exposed position on the virus surface and its crucial role in virus entry.^8^^,^^9^

MVA-MERS-S is one of the only three vaccine candidates that have reported results from early-phase clinical studies.^10^^,^^11^^,^^12^^,^^13^ Based on the Modified Vaccinia virus Ankara (MVA) vector, MVA-MERS-S encodes for the full-length S protein.^14^ The vaccine was shown to be safe and immunogenic in a phase 1 clinical trial conducted in healthy adults in Hamburg, Germany, including 23 study participants who received two vaccinations 28 days apart.^12^ A subgroup of ten study participants received an additional booster vaccination with MVA-MERS-S 12 ± 4 months after the first vaccination, which strongly enhanced titers and persistence of binding and neutralizing antibodies, as well as frequencies of memory B cells (MBCs).^15^^,^^16^

Binding and neutralizing antibodies have been shown to correlate with protection against many viral diseases and are commonly used in clinical vaccine trials to assess vaccine immunogenicity.^17^^,^^18^^,^^19^ However, among other discoveries related to vaccine-induced immune responses, it has become apparent that additional antibody features can contribute to protection against viral infection and/or disease. Numerous studies underlined an important role of Fc-mediated antibody functions such as antibody-dependent cellular cytotoxicity (ADCC), monocyte and neutrophil phagocytosis (ADCP, ADNP), as well as complement deposition (ADCD).^20^^,^^21^^,^^22^ In addition, structural antibody properties such as subclass and glycan modifications have been shown to strongly influence Fc functionality.^21^^,^^23^ Combining a comprehensive analysis of multiple antibody features with advanced statistical methods, systems serology aims to increase the insights from clinical studies and improve the understanding of vaccine-induced immune mechanisms.^24^^,^^25^

In the present study, we apply a systems serology approach to dissect humoral immune responses induced by vaccination with MVA-MERS-S. Based on the study cohort receiving two vaccinations in the primary series and a late booster, we conducted an exploratory analysis to gain detailed insight into structural and functional antibody properties, in addition to the previously published data on S-specific IgG and neutralizing antibodies.^16^ Using purified IgG from vaccinee plasma samples, we investigated antibody subclasses, *N*-linked Fc glycan composition, and capacities to mediate ADCP and antibody-dependent NK cell activation (ADNKA, a surrogate for ADCC). Supported by a comprehensive data analysis, our study underlines the potential of a late booster vaccination to enhance neutralizing and Fc-mediated antibody functionality and highlights the distinct characteristics of vaccine-induced antibodies specific to the MERS-CoV S protein subunits S1 and S2.

## Results

### A late homologous booster vaccination with MVA-MERS-S increases titers and functionality of MERS-CoV S-specific antibodies

**Figure 1:**
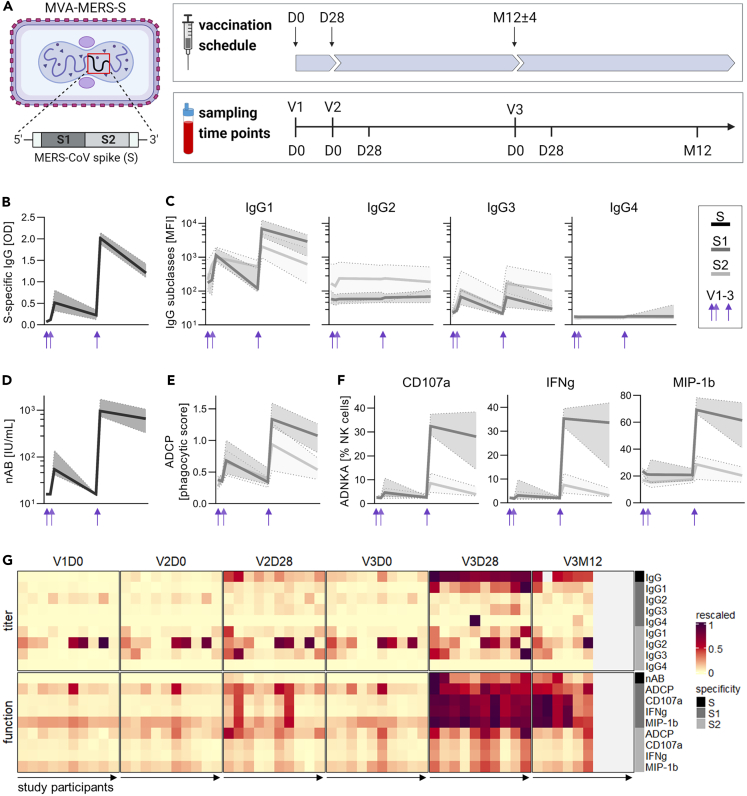
A late homologous booster vaccination with MVA-MERS-S increases titers and functionality of MERS-CoV-spike (S)-specific antibodies (A) Study design. Ten study participants received 3 vaccinations (V1, V2, V3) with MVA-MERS-S at day 0 (D0), day 28 (D28), and month 12 (M12). Blood was sampled longitudinally, at defined time points before and after each vaccination. Figure created with Biorender. (B-F) Dynamics of antibody titers and functionality specific to the MERS-CoV S protein and its subunits S1 and S2, measured at longitudinal time points indicated in panel (A). Shown are S-specific IgG (as reported previously^16^) (B), subclasses IgG1-IgG4 (C), neutralizing antibodies (nAB) (D), antibody-dependent cellular phagocytosis (ADCP) (E), and antibody-dependent NK cell activation (ADNKA), as indicated by activation markers CD107a, IFNγ and MIP-1β (F). Shown are the median and interquartile range (IQR). Individual values of each parameter are shown in Figures S1 and S2 and gating strategies for the evaluation of ADCP and ADNKA are depicted in Figure S3. See also Figure S4. Median, min-max range, and sample size are indicated in Tables S1 and S2. IgG, nAB, and ADNKA were measured as single replicates, while IgG1-4 were measured as duplicates and ADCP as triplicates. (G) Heatmap summarizing antibody titers (IgG, IgG1-4) and functionality (nAB, ADCP, ADNKA (CD107a, IFNγ, MIP-1β)), longitudinally. Each tile indicates an individual data point, rescaled for each parameter across all study participants and time points. Missing values are indicated by gray tiles. Specificities for whole S and subunits S1 and S2 are indicated by color blocks. OD: optical density; MFI: median fluorescence intensity.

As part of a phase 1 trial and follow-up study, a subgroup of ten study participants received three vaccinations (V1, V2, V3) with the MVA-MERS-S vaccine candidate. The primary vaccination series consisted of two immunizations given 28 days apart, followed by a late third immunization administered at 12 ± 4 months after the first vaccination. Antibodies were comprehensively characterized at multiple time points, including the baseline (day 0) of each vaccination (V1D0, V2D0, V3D0), as well as the peak response (day 28) after the second and third vaccination (V2D28, V3D28). Six participants were additionally included in a late follow-up visit (month 12) after the late boost (V3M12) (Figure 1A). Due to the small sample size, we did not perform statistical significance testing on our dataset, as will be further discussed in the limitations of the study.

In a first step, MERS-CoV S-specific IgG was analyzed by an enzyme-linked immunosorbent assay (ELISA) (Figures 1B and S1). After the completion of the primary vaccination series, S-specific IgG was induced in all ten study participants and decreased during the following months. Compared to the primary vaccination series, the late boost strongly increased the antibody titers, resulting in higher levels at peak response and enhanced persistence (median [IQR] (OD): V2D28 = 0.51 [0.32–0.81]; V3D28 = 2.01 [1.88–2.15]; V3M12 = 1.21 [1.1–1.42]), as reported previously.^16^ In the next step, we purified IgG from vaccinee plasma samples and used a bead-based multiplex immunoassay to further analyze the composition of IgG subclasses, as well as their specificity toward the S protein subunits S1 and S2 (Figures 1C and S1). For both S1- and S2-specific responses, IgG1 was the predominant subclass. S1-specific IgG1 responses showed an overall similar pattern as observed for total IgG, with an induction observed already after the primary vaccination series and a strong increase after the late boost, as well as persistence until month 12 post V3 (median [IQR] (MFI): V2D28 = 1144 [783–1799]; V3D28 = 6898 [3982–12337]; V3M12 = 2890 [1053–4538]). Compared to S1-specific responses, S2-specific IgG1 antibodies showed similar levels after the primary vaccination series, but revealed a limited boosting capacity and persistence following V3 (median [IQR] (MFI): V2D28 = 991 [687–1973]; V3D28 = 2053 [968–4998]; V3M12 = 612 [163–1836]). A quantitatively minor subset of IgG3 antibodies was observed for S2-, and to a lesser extent for S1-specific responses both after V2 (median [IQR] (MFI): S2, V2D28 = 109 [70–391]; S1, V2D28 = 67 [39–106]) and V3 (median [IQR] (MFI): S2, V3D28 = 166 [88–546]; S1, V3D28 = 66 [33–210]), whereas IgG2 and IgG4 were not induced by vaccination with MVA-MERS-S.

Besides binding antibody titers, we also assessed neutralizing and Fc-mediated antibody functionality. The analysis of neutralizing antibodies (nAB) by a pseudovirus neutralization assay revealed a strong increase of nAB after the late boost, which persisted during the following 12 months (median [IQR] (IU/mL): V2D28 = 55 [41–138]; V3D28 = 961 [736–1734]; V3M12 = 660 [325–1052]) (Figures 1D and S2). Based on the monocytic THP-1 cell line, we evaluated the capacity of purified S-specific IgG to mediate ADCP. The uptake of fluorescent beads, coupled with S-specific IgG antibodies, was measured via flow cytometry and quantified as a phagocytic score (Figures 1E, S2, and S3). The ADCP capacity of S1-specific IgG strongly increased after the late boost, relative to the primary vaccination series (median [IQR] (phagocytic score): V2D28 = 0.7 [0.5–1.0]; V3D28 = 1.3 [1.2–1.6]). As observed for IgG1, the ADCP capacity revealed a limited boosting effect for S2-specific compared to S1-specific responses. Compared to S1, S2-specific ADCP revealed similar responses after the primary vaccination series, but lower responses after V3 (median [IQR] (phagocytic score): V2D28 = 0.7 [0.4–0.8]; V3D28 = 0.9 [0.5–1.3]). To assess ADNKA functionality, NK cells were isolated from a healthy donor and stimulated with purified S-specific IgG from vaccinee plasma samples. NK cell activation was analyzed by the expression of CD107a, IFNγ and MIP-1β, as measured via flow cytometry (Figures 1F, S2, and S3). For S1-specific IgG, all three activation markers indicated a very limited ADNKA capacity after the primary vaccination series, which was strongly enhanced after the late boost (median [IQR] (% NK cells): CD107a, V2D28 = 4.7 [3.0–10.6]; CD107a, V3D28 = 32.5 [28.9–37.5]; IFNγ, V2D28 = 3.2 [1.7–9.7]; IFNγ, V3D28 = 35.3 [32.0–39.5]; MIP-1β, V2D28 = 21.1 [15.1–32.2]; MIP-1β, V3D28 = 69.2 [66.3–78.4]). In comparison, the ADNKA capacity of S2-specific IgG remained low after all three vaccinations (median [IQR] (% NK cells): CD107a, V2D28 = 3.6 [2.3–6.4]; CD107a, V3D28 = 8.5 [4.7–12.7]; IFNγ, V2D28 = 3.0 [1.5–4.7]; IFNγ, V3D28 = 7.5 [4.2–12.2]; MIP-1β, V2D28 = 15.4 [11.5–21.2]; MIP-1β, V3D28 = 28.7 [17.6–34.9]).

All analyzed humoral immune parameters are summarized in Figure 1G according to time point, parameter, and individual. The heatmap underlines the impact of the late booster vaccination which leads to a strong increase of several parameters, including S-specific IgG and IgG1 as well as neutralizing and Fc-mediated functionality. In addition, the heatmap highlights the differences between S1- and S2-specific antibody responses. Overall, S2-specific responses showed a stronger variability between study participants, both at baseline and post-vaccination. While S1-specific IgG1, ADCP, and ADNKA showed a stronger induction after the late boost compared to the primary vaccination, this effect was less pronounced for S2-specific responses.

Notably, MVA-MERS-S vaccination did not increase antibody levels against the human coronaviruses (HCoV)-OC43, -NL63 and -HKU1, measured by protein microarray (PMA), as reported by Raadsen et al.^26^ A correlation analysis of pre-existing HCoV-specific antibodies (V1D0) with vaccine-induced MERS-CoV-specific antibodies (V2D28) revealed no substantial correlations (|r| < 0.4) (Figure S4).

### S2-specific parameters reveal positive correlations after the late boost, while relationships between S1-specific parameters are more divers

**Figure 2:**
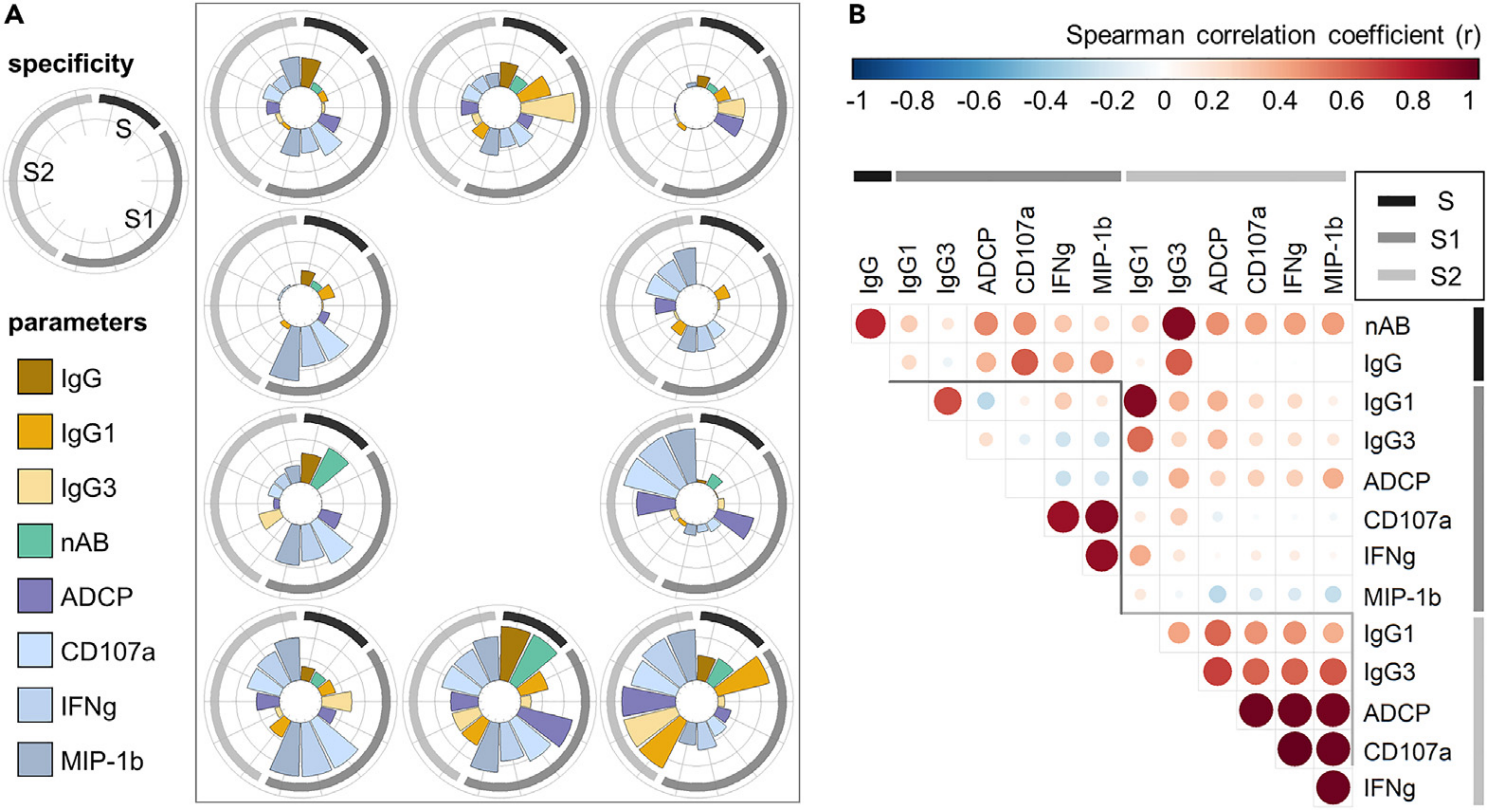
S2-specific parameters reveal positive correlations after the late boost, while relationships between S1-specific parameters are more divers (A) Circular bar plots depicting individual immune signatures for each of the ten study participants at 28 days after the third vaccination (V3D28). Parameters are indicated by bar colors, and antigen specificity toward whole spike (S) protein and subunits S1 and S2 by outer circular legend. Data from V3D28 was rescaled for each parameter and specificity, across all study participants. (B) Spearman correlation matrix of immune parameters at V3D28. Antibody specificities are indicated by color blocks. Color and size of the circle indicate the correlation coefficient (r).

Next, we analyzed the relation between different antibody parameters induced by the late booster vaccination in more detail, focusing on V3D28 and taking into account only parameters with a relevant induction over time. Humoral immune signatures of each study participant are displayed as circular bar plots of rescaled parameters, highlighting disparities in individual response patterns (Figure 2A). Notably, this kind of visualization aims at a comparison of the relative value of each individual within the range of each parameter, while it is not suitable to compare absolute values between parameters. A correlation analysis revealed different correlation patterns for S1- and S2-specific parameters (Figure 2B). For S2-specific responses, positive correlations were observed between all measured parameters. The strongest correlations (r ≥ 0.95) were detected between all markers for the Fc functionality, namely ADCP and ADNKA (CD107a, IFNγ, MIP-1β). Moderate correlations were detected between IgG1/IgG3 titers and Fc-dependent functions, with overall stronger correlations for IgG3 (r = 0.59 to 0.68) compared to IgG1 (r = 0.36 to 0.58). For S1-specific responses, the strongest correlations were observed between the three markers representing ADNKA (r ≥ 0.85). However, as opposed to S2-specific responses, these markers did not correlate with ADCP, and no correlations were observed between Fc-mediated functions and IgG1/IgG3 titers (|r| ≤ 0.26). The correlations between the two antigen specificities (S1, S2) revealed to be mostly low or non-existent (|r| ≤ 0.37). However, strong to moderate correlations were observed for S2-specific IgG1 with S1-specific IgG1 and IgG3, respectively (r = 0.92; r = 0.56). nABs showed the strongest correlation with S2-specific IgG3 (r = 0.92), followed by IgG (r = 0.76). S-specific IgG showed the strongest correlations with S1-specific ADNKA (CD107a) and S2-specific IgG3 (r = 0.60).

### S1- and S2-specific IgG1 possess differential compositions of *N*-linked Fc glycans

**Figure 3:**
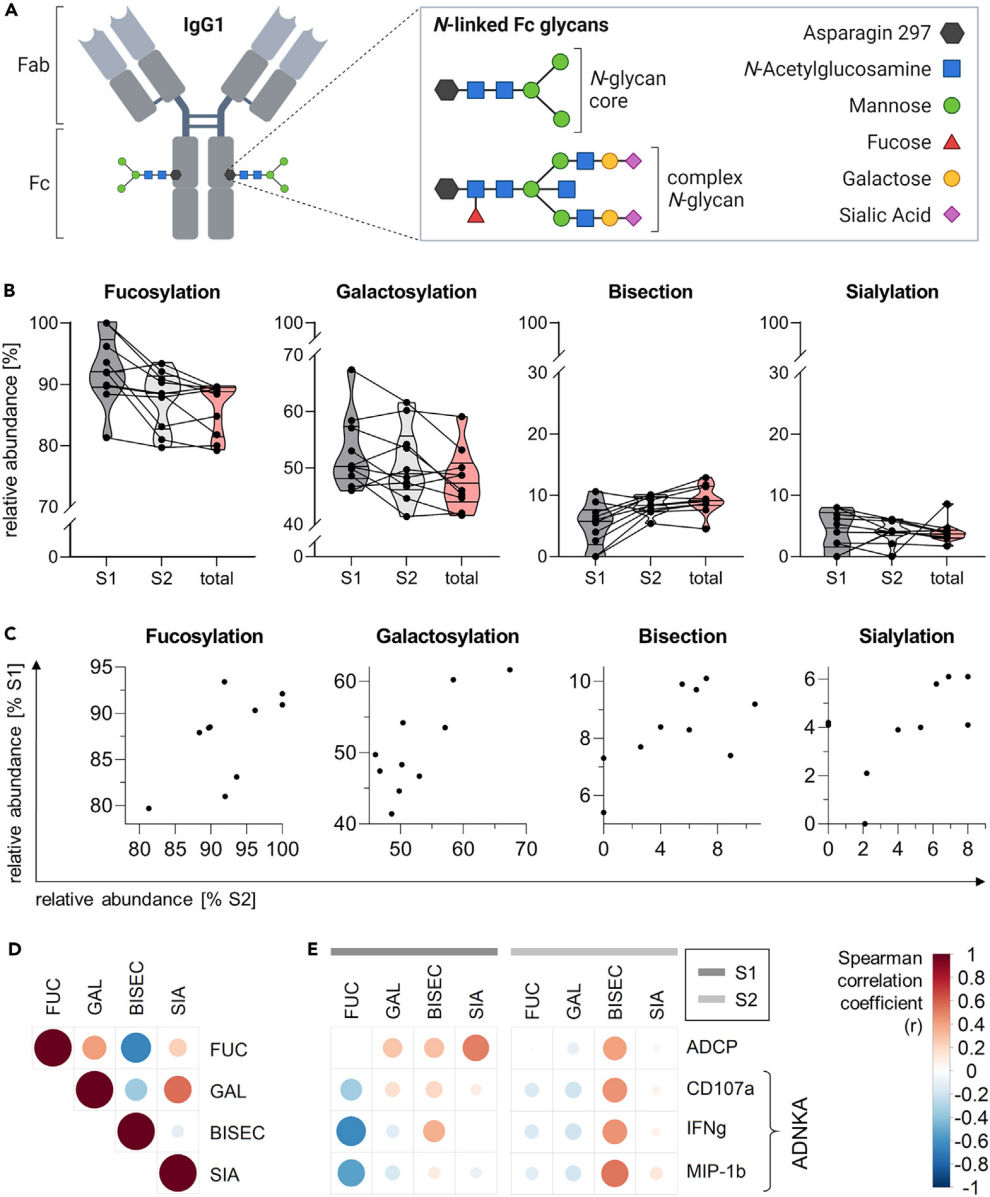
S1- and S2-specific IgG1 possess differential compositions of *N*-linked Fc glycans (A) Schematic of IgG1 molecule with *N*-linked Fc glycans, figure created with Biorender. (B) Relative abundance of major glycosylation traits within *N*-linked Fc glycans of MERS-CoV spike (S) S1/S2-specific and total (unspecific) IgG1 at 28 days after the third vaccination (V3D28). Median, quartiles, and min-max ranges are indicated by violin plots. Individual values are plotted as dots and connecting lines indicate samples belonging to the same study participant. Median, min-max range, and sample size are indicated in Table S5. (C) Spearman correlations between glycosylation traits of S1- and S2-specific IgG1 Fc glycans. (D) Spearman correlation matrix of glycosylation traits, based on pooled data of S1- and S2-specific IgG1. (E) Spearman correlation matrix of IgG1 glycosylation traits and IgG Fc functionality in the S1- (left panel) and S2-specific (right panel) antibody compartment. (D and E) Color and size of the circle indicate the correlation coefficient (r). FUC: fucosylation, GAL: galactosylation, BISEC: bisection, SIA: sialylation, ADCP: antibody-dependent cellular phagocytosis; ADNKA: antibody-dependent NK cell activation.

To gain further insight into structural antibody properties, we analyzed the composition of *N*-linked glycans attached to the Fc region of total (unspecific), S1- and S2-specific IgG1 antibodies (schematic shown in Figure 3A), using mass spectrometry. Across all samples, 18 different glycan species were identified (Tables S3 and S4), and summed up into four major glycosylation traits: fucosylation, galactosylation, bisection, and sialylation (Figure 3B, Table S5). At time point V3D28, the glycan composition tended to differ between S1- and S2-specific IgG1, whereas S2-specific IgG1 seemed to have a more similar glycan distribution to total IgG1. Specifically, fucosylation of S1-specific IgG1 tended to be enhanced compared to S2-specific and total IgG1 (median frequency (mfr) [IQR] (%): S1 = 92.0 [89.4–97.2], S2 = 88.5 [82.6–91.2], total = 88.7 [81.4–89.4]) and in tendency, the same pattern was observed for galactosylation (mfr [IQR] (%): S1 = 50.3 [48.1–57.4], S2 = 49 [46.2–55.7], total = 47.4 [44.0–50.9]). Bisection was reduced in S1-specific IgG1 compared to the other groups (mfr [IQR] (%): S1 = 5.8 [2.0–7.6], S2 = 8.4 [7.4–9.8], total = 9.1 [8.4–11.5]), while sialylation showed a more heterogeneous pattern (mfr [IQR] (%): S1 = 4.7 [1.6–7.2], S2 = 4.1 [3.5–5.9], total = 3.7 [3.1–4.3]). Notably, positive correlations (r = 0.50 to 0.65) were observed between S1- and S2-specific IgG1 for all four glycosylation traits (Figure 3C). A positive correlation was additionally observed between S1/S2-specific galactosylation and sialylation (r = 0.55), whereas fucosylation correlated negatively with bisection (r = −0.65) (Figure 3D). A correlation analysis of the glycosylation traits with the ADCP and ADNKA functionality revealed negative correlations between fucosylation and ADNKA (r = −0.34 to −0.63) and a positive correlation between sialylation and ADCP (r = 0.51) in the S1-specific antibody compartment. Positive correlations between bisection and ADCP/ADNKA were observed for S2-specific antibodies (r = 0.41 to 0.54) (Figure 3E).

## Discussion

In the present study, we applied an exploratory systems serology approach to gain comprehensive insight into humoral immune responses induced by the MVA-MERS-S vaccine candidate. While previous studies revealed increased titers and persistence of binding and neutralizing antibodies after a late boost with MVA-MERS-S, administered approximately one year after prime,^15^^,^^16^ we here report on the enhanced induction of Fc-mediated, non-neutralizing antibody functions, based on the same study cohort. For binding and neutralizing antibodies, enhanced induction after late booster vaccination has been reported in various vaccine trials, including studies on MVA-based vaccines, whereas its effect on non-neutralizing antibody functions remains less well studied.^27^^,^^28^^,^^29^ In our study, both ADCP and ADNKA capacity increased after the late boost compared to the primary vaccination series, in line with findings reported in the context of vaccination against SARS-CoV-2 and HIV-1. In a study by Tong et al.,^30^ a late booster vaccination with a SARS-CoV-2 mRNA vaccine after approximately seven months yielded higher levels of ADCP, ADNKA, ADNP, and ADCD compared to a two-dose primary vaccination series with either an mRNA or inactivated SARS-CoV-2 vaccine. Enhanced ADCP, ADNKA, ADNP, and ADCD capacities were also observed after an additional late boost with a viral vector and/or subunit vaccine at twelve months in the RV144 HIV vaccine trial, using a heterologous prime-boost strategy.^31^

The functionalities of both the Fab and Fc antibody regions are known to be affected by maturation processes that are induced in B cells during the germinal center (GC) reaction, namely affinity maturation and isotype switching.^32^^,^^33^ The increased immunogenicity observed after late booster vaccination is likely affected not only by the administration of an additional vaccine dose but also by the prolonged interval between doses, supporting the generation of mature MBCs that are able to give rise to antibodies of increased quantity and quality upon reactivation.^18^^,^^34^^,^^35^ Notably, a strong recall response of MBCs has been previously reported after the late boost with MVA-MERS-S in this trial,^16^ and might be related to the increase of antibody titers and functionality. While the effects of an additional vaccination and a prolonged immunization interval cannot be distinguished with our data, a study by Kreijtz et al.*,*^27^ investigating an MVA-based H5N1 vaccine, showed that a late booster vaccination one year after prime induced similar levels of neutralizing antibodies in two cohorts receiving either one or two doses as primary vaccination series. For the MVA-MERS-S vaccine, a subsequent clinical trial is currently underway, investigating two different intervals (28 or 56 days) between the first and second vaccination (ClinicalTrials.gov: NCT04119440). In that study, higher levels of MERS-CoV-specific antibodies were induced in the 56- compared to the 28-day interval group.^36^

The S1 subunit of the MERS-CoV S protein is often considered the most important target of neutralizing antibodies, as it contains the receptor binding domain (RBD) responsible for binding to the host cell receptor dipeptidylpeptidase 4 (DPP4).^37^ However, neutralizing activity and Fc functionality have also been described for antibodies directed against S2, and their importance has recently been discussed in the context of both MERS-CoV and SARS-CoV-2.^38^^,^^39^^,^^40^^,^^41^^,^^42^^,^^43^^,^^44^^,^^45^ The S2 subunit, which mediates the fusion of virus and host cell membranes, is more conserved among different species of HCoV and has been shown to be less prone to changes in SARS-CoV-2 variants.^46^^,^^47^^,^^48^^,^^49^ It has therefore been suggested as an antigen for pan-coronavirus vaccines, analogous to the approach of using the conserved stem region of the hemagglutinin protein for the development of a universal Influenza vaccine.^42^^,^^48^^,^^50^^,^^51^^,^^52^^,^^53^ S2-specific antibodies that are cross-reactive between different HCoV strains have been described by several studies, but their protective role remains elusive.^46^^,^^54^^,^^55^^,^^56^^,^^57^^,^^58^^,^^59^

In our study, we observed differential dynamics of S1- and S2-specific antibodies, with overall similar induction after the primary vaccination series, but a stronger boosting capacity of S1-specific responses following the late boost. One explanation for the higher boosting capacity of S1-specific responses could be a higher intrinsic immunogenicity of the S1 subunit.^18^^,^^60^^,^^61^ It could also be speculated that the limited boosting capacity of S2-specific responses might be partially caused by a phenomenon referred to as *immune imprinting* or *original antigenic sin*: As MBCs enter the GC reaction more easily compared to naive B cells, cross-reactive S2-specific MBCs from previous HCoV infections might have an advantage in the selection for the GC reaction compared to S2-specific naive B cells, but could then be limited in proliferation and maturation processes due to their reduced affinity to the slightly different S2 antigen from MERS-CoV.^59^^,^^62^^,^^63^^,^^64^^,^^65^ Notably, pre-existing antibodies against HCoV-OC43, HCoV-NL63 and HCoV-HKU1^15^ did not show substantial correlations with vaccine-induced MERS-CoV-specific antibodies in our study.

Besides the differential dynamics of S1- and S2-specific antibody titers, our study suggests S1-specific IgG antibodies to have a higher *in vitro* ADCP and ADNKA capacity after the late boost. Fc functionality can be influenced by multiple factors, including subclass and Fc glycosylation.^23^^,^^66^ As expected for vaccination with a viral vector encoding a protein antigen,^23^ MVA-MERS-S mainly induced antibodies of the IgG1 and IgG3 subclasses. IgG1 and especially IgG3 have been associated with a high functional capacity in the past,^67^ which is supported by positive correlations within the S2-specific antibody compartment in our study. The lack of fucosylation (afucosylation) and the bisection of *N*-linked Fc glycans have been shown to increase the IgG binding affinity for the Fc gamma receptor III (CD16), and thereby enhance the capacity to induce ADCC.^68^^,^^69^^,^^70^ In line with this, we observed negative correlations for the fucosylation of S1-specific antibodies and positive correlations for the bisection of S2-specific antibodies, respectively, with their ADNKA capacity. However, these findings have to be interpreted carefully due to the small sample size and the Fc functionality not being normalized for the titers of S1/S2-specific IgG antibodies. Methodological aspects such as the assay format and antigen conformation can additionally impact functional antibody readouts. Furthermore, the overall IgG functionality *in vivo* can be influenced by additional factors including the presence of other antibody isotypes (IgM, IgA) competing for antigen binding or interacting with their cognate Fc receptors, thus shaping the resulting effector mechanisms.^71^^,^^72^

The sampling time point can strongly impact the *N*-linked glycan composition as underlined by Collie et al., reporting a transient afucosylation of S-specific IgG1 after primary vaccination which decreased rapidly within two to three weeks.^73^ Notably, S2-specific antibodies overall resembled the glycan pattern of total IgG1 in our study, whereas S1-specific IgG1 displayed a more distinct glycan composition, indicating a more pronounced vaccine-induced effect. In line with previous studies,^74^^,^^75^ an inverse correlation was observed between bisection and fucosylation, whereas galactosylation and sialylation correlated positively. Positive correlations for all four glycosylation traits between S1- and S2-specific IgG1 may indicate a conserved glycan pattern for each study participant.

Correlates of protection have not yet been defined for MERS-CoV, and whether the antibodies induced by MVA-MERS-S vaccination are protective against MERS-CoV infection and/or disease, cannot be answered by our study. However, several studies have investigated monoclonal antibodies isolated e.g., from MERS convalescent patients, revealing a protective capacity of neutralizing antibodies upon MERS-CoV challenge in different animal models.^47^^,^^76^^,^^77^^,^^78^^,^^79^ In the context of SARS-CoV-2 infection and vaccination, non-neutralizing Fc-mediated antibody functions such as ADCC and ADCP have been suggested to additionally contribute to protection from COVID-19, in particular against SARS-CoV-2 variants.^19^^,^^80^^,^^81^^,^^82^^,^^83^^,^^84^^,^^85^ While neutralizing antibodies are thought to be important to block virus transmission in the first place, Fc-mediated antibody functions likely contribute to virus clearance during the course of infection.^25^^,^^84^^,^^86^

Taken together, our exploratory study highlights the benefit of a late homologous booster vaccination with MVA-MERS-S to enhance not only antibody titers and persistence but also neutralizing and Fc-mediated, non-neutralizing antibody functionality. Additionally, our systems serology analysis gives important insight into the distinct characteristics of antibodies directed against the S protein subunits S1 and S2, which might be of relevance for the future design of HCoV vaccines.

### Limitations of the study

A limitation of our study is the small size of the study cohort. However, relatively small sample sizes have been inherent to exploratory systems serology studies requiring deep immunological assessments, and our study has the strength of repeated longitudinal sampling in the same volunteers. Due to the limited sample size and the multiple immune parameters evaluated, resulting in a high dimensional dataset, we did not perform any statistical significance testing as the interpretability of *p*-values would be hindered by reduced power and multiple testing issues. In addition, we were not able to evaluate the influence of factors such as sex and ethnicity, as all study participants were female and white. While we here focused on IgG which was previously shown to be the isotype predominantly induced by MVA-MERS-S vaccination,^16^ IgM and IgA isotypes may also impact the overall antibody functionality, which was not assessed in our study. Despite its exploratory nature in a small cohort, our study is the first to apply systems serology in the context of a MERS vaccine and provides insights for further studies.

## STAR★Methods

### Key resources table

**Table:** 

REAGENT or RESOURCE	SOURCE	IDENTIFIER
**Antibodies**		

Rabbit anti-MERS-CoV Nucleoprotein antibody	Genetex	Cat#GTX134868; RRID: AB_2887364
Goat anti-Rabbit IgG antibody, Alexa Fluor 488	Thermo Fisher Scientific	Cat#A32731; RRID: AB_2633280
Mouse Anti-Human IgG1 Hinge-PE	SouthernBiotech	Cat#9052-09; RRID: AB_2796621
Mouse Anti-Human IgG2 Fc-PE	SouthernBiotech	Cat#9070-09; RRID: AB_2796639
Mouse Anti-Human IgG3 Hinge-PE	SouthernBiotech	Cat#9210-09; RRID: AB_2796701
Mouse Anti-Human IgG4 Fc-PE	SouthernBiotech	Cat#9200-09; RRID: AB_2796693
Mouse anti-VSV (monoclonal), clone 8G5F11	Absolute Antibody	Cat#EB0010; RRID: AB_2811223
WHO international human MERS-CoV IgG standard	NIBSC	Cat#19/178
PE anti-human CD107a (LAMP-1) Antibody	Biolegend	Cat#328608; RRID: AB_1186040
Brilliant Violet 785™ anti-human CD56 (NCAM) Antibody	Biolegend	Cat#362550; RRID: AB_2566059
Brilliant Violet 510™ anti-human CD16 Antibody	Biolegend	Cat#302048; RRID: AB_2562085
APC/Cyanine7 anti-human CD3 Antibody	Biolegend	Cat#344818; RRID: AB_10645474
APC/Cyanine7 anti-human CD14 Antibody	Biolegend	Cat#367107; RRID: AB_2566710
APC/Cyanine7 anti-human CD19 Antibody	Biolegend	Cat#363010; RRID: AB_2564193
PE/Cyanine7 anti-human IFN-γ Antibody	Biolegend	Cat#506518; RRID: AB_2123321
BD Horizon™ BV421 Mouse Anti-Human MIP-1β	BD Biosciences	Cat#562900; RRID: AB_2737877

**Bacterial and virus strains**		

Recombinant Vesicular Stomatitis Virus lacking G protein, GFP tagged	Mykytyn et al.^87^	VSV-ΔG-GFP

**Biological samples**		

Cryopreserved plasma from MVA-MERS-S vaccinees	University Clinical Center Hamburg-Eppendorf, Germany	N/A
Cryopreserved serum from MVA-MERS-S vaccinees	University Clinical Center Hamburg-Eppendorf, Germany	N/A

**Chemicals, peptides, and recombinant proteins**		

Recombinant clamp MERS-CoV-spike protein	Keith Chappell, The School of Chemistry and Molecular Biosciences, University of Queensland, Brisbane, QLD, Australia	N/A
MERS-CoV Spike Protein (S1 Subunit, aa 1-725, His Tag)	Sino Biological	Cat#40069-V08B1
MERS-CoV Spike Protein (S2 Subunit, aa 726-1296, His Tag)	Sino Biological	Cat#40070-V08B
LIVE/DEAD™ Fixable Violet Dead Cell Stain Kit	Thermo Fisher Scientific	Cat#L34964
Zombie NIR™ Fixable Viability Kit	Biolegend	Cat# 423106
Opti-MEM I (1×) + GlutaMAX	Gibco	Cat#51985-042
Apoptosis inhibitor Y27632	MedChemExpress	Cat# HY-10583
Hoechst 33342	Life technologies	Cat# 62249
MagPlex-C Microspheres	Luminex	Cat#MC10012-01, Cat#MC10015-01
FluoSpheres™ NeutrAvidin™-Labeled Microspheres	Thermo Fisher Scientific	Cat#F8776
Protein A Dynabeads	Thermo Fisher Scientific	Cat#10001D
Sequencing Grade Modified Trypsin	Promega	Cat#V511A
Peptide Calibration Standard II	Bruker Daltonics	Part No: 8222570
α-Cyano-4-hydroxycinnamic acid	Bruker Daltonics	Part No: 8201344

**Deposited data**		

Mass spectrometry/glycoproteomics data	This paper	GlycoPOST(https://glycopost.glycosmos.org/): GPST000440.0

**Experimental models: Cell lines**		

HEK-293T	ATCC	Cat#CRL-3216
Calu-3	ATCC	Cat#HTB-55
THP-1	ATCC	Cat#TIB-202
Primary NK cells isolated from healthy donor	University Clinical Center Hamburg-Eppendorf, Germany	N/A

**Recombinant DNA**		

pCAGGS encoding MERS-coronavirus Spike protein (EMC/2012)	This study	pCAGGS-MERS-S

**Software and algorithms**		

Bio-Plex Manager™ Software v6.2 (build 175)	Bio-Rad Laboratories, Inc.	https://www.bio-rad.com/
Harmony version 4.9	Perkin Elmer	https://www.perkinelmer.com/
GraphPad Prism version 9.5.1/10.0.3	Dotmatics	https://www.graphpad.com/
SpectroFlo® v3.0.1	Cytek Biosciences	https://cytekbio.com/
FACSDiva Software v8.0.1	BD Biosciences	https://www.bdbiosciences.com/
FlowJo v.10.7.2	FlowJo, LLC	https://www.flowjo.com/
Compass 2.0 flexSeries (flexcontrol & flexanalysis)	Bruker Daltonics	Material No: 1830951
R v. 4.3.2	The R Foundation	https://www.r-project.org/foundation/
RStudio 2023.12.0 + 369	Posit PBC	https://posit.co/

### Resource availability

#### Lead contact

Further information and requests for resources and reagents should be directed to and will be fulfilled by the lead contact, Leonie M. Weskamm (m.weskamm@uke.de).

#### Materials availability

This study did not generate new unique reagents.

#### Data and code availability

• De-identified human mass spectrometry/glycoproteomics data (*N*-linked Fc glycan analysis) have been deposited in GlycoPOST.^88^ They are publicly available as of the date of publication. Accession numbers are listed in the key resources table. All other data reported in this paper will be shared by the lead contact upon request.
• This paper does not report original code.
• Any additional information required to reanalyze the data reported in this paper is available from the lead contact upon request.

### Experimental model and study participant details

#### Vaccine construct

As reported previously in more detail,^12^^,^^14^^,^^15^^,^^16^ MVA-MERS-S is based on a recombinant MVA vector encoding the full-length MERS-CoV S-glycoprotein, based on the sequence of EMC/2012 (GenBank: JX869059). The vaccine was manufactured by IDT Biologika GmbH (Dessau, Germany) in primary chicken embryo fibroblasts (CEF).

#### Study design and participants

The first in-human study of the MVA-MERS-S vaccine candidate was conducted between 2017 and 2020 at the University Medical Center Hamburg-Eppendorf (UKE) in Hamburg (Germany). This phase 1 clinical trial addressed safety and immunogenicity of MVA-MERS-S in healthy adults, aged between 18 and 55. Study participants received two immunizations with either 1×10^7^ plaque-forming units (PFU, low dose) or 1 × 10^8^ PFU (high dose), 28 days apart.^12^ A subgroup of ten study participants from the low dose (*n* = 3) and the high dose (*n* = 7) groups received a late booster immunization of 1 × 10^8^ PFU (high dose) 12 ± 4 months after prime immunization, and six participants were followed up for another two years after the late booster vaccination.^15^^,^^16^ This subgroup (*n* = 10) represents the study population of this article, with the following baseline characteristics: age = 18–40 years (median = 29); sex = 100% female; ethnicity = 100% white. The study design of the phase 1 clinical trial was reviewed and approved by the Competent National Authority (Paul-Ehrlich-Institut, PEI, Langen, Germany) and the Ethics Committee of the Hamburg Medical Association, and is registered at ClinicalTrials.gov: NCT03615911. The observational study for the late follow-up time points was approved by the Ethics Committee of the Hamburg Medical Association and is registered under Protocol No. PV6079. Both studies were performed in accordance with the Declaration of Helsinki in its version of Fortaleza 2013. All participants provided written informed consent prior to enrollment in the studies.

#### Blood sampling

Serum and EDTA blood were sampled from all study participants (*n* = 10) before vaccination 1 (V1D0), at days 0 and 28 after vaccination 2 (V2D0, V2D28) and at days 0 and 28 after vaccination 3 (V3D0, V3D28). Additionally, blood was sampled from a subgroup of study participants (*n* = 6) between days 325 and 347 (12 months) after V3 (V3M12). Plasma was isolated from EDTA blood via centrifugation at 1000*g* for 15 min. Plasma and serum samples were stored at −80°C.

#### IgG purification from plasma samples

IgG was purified from vaccinee plasma samples using the Melon Gel IgG Spin Purification Kit (ThermoScientific, 45206) according to the manufacturer’s instructions.

#### Cell lines and primary cultures

Monocytic THP-1 cells (ATCC, originally obtained from male human subject) for the ADCP assay were maintained in RPMI-1640 medium supplemented with 10% FBS, at 37°C and 5% CO_2_. Primary NK cells for the ADNKA assay were isolated from EDTA blood of a healthy female donor, using the RosetteSep Human NK Cell Enrichment Cocktail (STEMCELL Technologies) according to the manufacturer’s instructions. Blood collection from this healthy donor was approved by the Competent National Authority (Paul-Ehrlich-Institut, Langen, Germany) and the Ethics Committee of the Medical Association Hamburg, Germany (reference numbers: 2020-10376-BO-ff, PV4780).

### Method details

#### ELISA

Total MERS-CoV S-specific IgG was measured using an in-house indirect ELISA, as described previously.^16^ Briefly, microplates were coated with 0.1 μg per well of full-length recombinant clamp MERS-CoV S protein (1 mg/mL, supplied by Keith Chappell, University of Queensland). After blocking, 100 μL of diluted serum (1:100) were incubated on the coated plates at 37°C for 60 min. Antibody staining was performed using horseradish peroxidase (HRP)-conjugated rabbit-*anti*-human IgG secondary antibody (Dako). The enzymatic color reaction was started by addition of 3,3′,5,5′-Tetramethylbenzidine (TMB) substrate and stopped by addition of 0.25 M sulfuric acid. The photometric readout was performed using the Tecan Infinite F200 microplate reader at a measurement wavelength of 450 nm with a reference wavelength of 620 nm. Results were reported as optical density (OD) values of the measurement minus reference wavelength and the cut-off OD value for positivity was set at > 0.1 above the geometric mean of negative control sera plus 3 standard deviations (0.094).

#### Bead-based multiplex immunoassay

A bead-based multiplex immunoassay was used to determine the proportion of subclasses within MERS-CoV S-specific IgG antibodies. This assay was based on purified IgG samples instead of whole plasma samples, as reported previously.^16^ Two regions of carboxylated microspheres (Luminex) were covalently coupled with either the S1 or S2 subunit of MERS-CoV S (Sinobiological) as described previously.^89^ Microspheres of both regions were then diluted to 50,000/mL in Assay Buffer (Merck KGaA) and added to a black, clear-bottom 96 well microplate in a volume of 50 μL (Greiner Bio-One). IgG samples were added at a volume of 50 μL (purified from plasma, followed by 1:10 dilution) and wells were filled up to 100 μL with Assay Buffer. Plates were incubated on an orbital shaker for 2 h at room temperature (RT) and 650 rpm. PE-conjugated detection antibodies specific for human IgG1, IgG2, IgG3 and IgG4 (Southern Biotech) were diluted to 0.65 μg/mL and 100 μL were added to respective wells for detection of microsphere-bound plasma antibodies. Plates were incubated on an orbital shaker for 2 h at RT and 650 rpm, and analyzed with a Bio-Plex 200 System. All measurements were performed in duplicates and the mean of both wells was used for further analysis.

#### Pseudovirus neutralization assay

While nAB titers of this study cohort were previously published as analyzed by a whole virus plaque reduction neutralization test (PRNT),^16^ they were here measured using a normalized MERS-CoV pseudovirus 50% focus reduction neutralization test (nFRNT_50_). Due to a continuous instead of an ordinal scale, the nFRNT data is more suitable for our systems approach including e.g., correlation analyses. Briefly, MERS-CoV S protein pseudotyped vesicular stomatitis virus (VSV) with Green Fluorescence Protein (GFP) reporter gene was generated as published previously.^87^ A monolayer of Human Embryonic Kidney (HEK)-293T cells was transfected with an eukaryotic expression vector (pCAGGs) containing a codon-optimized MERS-CoV S protein coding sequence, strain EMC/2012 (GenBank: JX869059). The following day, the cells were infected with VSV-ΔG-GFP, at a multiplicity of infection of 1–3. After 4 h of incubation, the cells were washed with medium and anti-VSV-G neutralizing antibody (clone 8G5F11; Absolute Antibody) was added to render any VSV-ΔG-GFP particles not pseudotyped with MERS-CoV S protein non-infective. Supernatant containing MERS-CoV pseudovirus was collected after 24 and 48 h, cleared by centrifugation and purified by ultracentrifugation on a 10% sucrose cushion. The resulting batch was titrated using 10-fold serial dilutions on Vero E6 cells (2x10^4^ per well) in 96 well plates. The foci were counted using image analysis software (Harmony version 4.9, Perkin Elmer). The highest dilution with detectable foci was quantified and multiplied by the dilution factor to obtain the number of infectious virus particles (focus forming units, FFU) per mL. The titer was determined based on the mean value of three replicates. MERS-CoV S protein expression was confirmed by western blot. Aliquots were snap frozen in a mixture of 100% ethanol and dry ice and stored at −80°C until use.

The nFRNT_50_ assay was performed on Calu-3 cells, grown to a confluent monolayer in 96-well flat-bottom cell culture plates (Greiner cat. No. 655180) in Opti-MEM, supplemented with 10% fetal bovine serum, penicillin and streptomycin (P/S, 100 IU/mL each) and Y-27632 apoptosis inhibitor (10 mM, MedChemExpress, Cat. No. HY-10583). Sera were heat-inactivated at 56°C for 30 min, and diluted in a 96-well V-bottom plate (Greiner cat. No. 655181) in a range of 1:20 to 1:14580 (3-fold, 7 times down) in Opti-MEM with P/S. A standard from the National Institute for Biological Standards and Control (NIBSC) was included on each plate, in a dilution range of 1:60 to 1:43740. Subsequently, 600 FFU of MERS-CoV pseudovirus were added per well, and the virus-serum mixture was incubated in a humidified CO_2_ incubator for 60 min at 37°C. Cells were washed once with serum-free Opti-MEM with P/S, prior to transfer of virus-serum mixture to the monolayer. After overnight incubation, cells were fixed in 4% paraformaldehyde and cell nuclei were stained with Hoechst dye and imaged using an automated confocal microscope (Opera Phenix, Perkin Elmer), followed by automated quantification of GFP-expressing cells using the Harmony software. A 50% reduction of GFP-expressing cells was considered to be neutralizing. Non-neutralizing serum samples were given a titer of 7, whereas nFRNT_50_ titers were calculated using 5-parameter logistic regression. Finally, sample titers were normalized by dividing them by the titer of the standard included on the same plate and multiplying the result by 1000 IU/mL, the unit assigned by NIBSC. Infectious VSV particles were handled in a laminar airflow cabinet under biosafety level II conditions.

#### ADCP assay

The ADCP capacity of MERS-CoV S-specific antibodies was measured using the monocytic THP-1 cell line and a flow cytometric readout. Briefly, MERS-CoV S protein S1 and S2 subunits (Sinobiological) were biotinylated using the EZ-Link Micro Sulfo-NHS-LC Biotinylation Kit (Thermo Fisher Scientific) according to the manufacturer’s instructions. Biotinylated S1 and S2 were then coupled to yellow-green fluorescent beads (FluoSpheres NeutrAvidin-Labeled Microspheres, Thermo Fisher Scientific) by incubation on an end-over-end rotational mixer at 4°C and 12 rpm overnight. Beads were washed and diluted 1:100 in PBS/0.1% BSA, referring to the original volume of beads used in the coupling reaction, and stored up to one week at 4°C. For the assay, 10 μL antigen-coupled fluorescent beads and 10 μL of IgG samples (purified from plasma, followed by 1:10 dilution) were added to a round bottom 96-well plate and incubated at 37°C and 5% CO_2_ for 2 h. After washing of the beads, THP-1 cells were added at a concentration of 1.25x10^5^/mL in 200 μL RPMI-1640 medium (ATCC) per well, followed by incubation at 37°C and 5% CO_2_ for 4 h. Cells were then stained with 100 μL staining buffer (PBS + 1% FBS, 1 mM EDTA) containing 0.1 μL viability stain (LIVE/DEAD Fixable Violet Dead Cell Stain Kit, Thermo Fisher Scientific) for 15 min at RT and subsequently fixed in 4% paraformaldehyde. The cells were analyzed by flow cytometry, using an LSR Fortessa (BD Biosciences). Based on the population of single, living THP-1 cells, the phagocytosis of beads was assessed via the yellow-green fluorescent emission in the FITC channel (for gating strategy see Figure S3). The ADCP capacity of the IgG samples was quantified as a phagocytic score, based on the percentage of FITC-positive cells (reflecting the percentage of cells with internalized beads) and the mean fluorescence intensity (MFI) of FITC-positive cells (reflecting the number of internalized beads per cell): Phagocytic score = % (FITC^+^ cells) x MFI (FITC^+^ cells)/1000. All measurements were performed in triplicates and the mean of the three wells was used for further analysis. For triplicates with a coefficient of variation >0.25, the replicate with the highest deviation was excluded from further analysis.

#### ADNKA assay

The ADNKA capacity of S-specific antibodies was measured using primary NK cells from healthy donors and a flow cytometric readout. Protein binding plates (Maxisorp, Sigma-Aldrich) were coated with 100 μL MERS-CoV S protein S1 and S2 subunits (Sinobiological) at a concentration of 5 μg/mL for 2 h at RT, followed by blocking with PBS containing 5% low IgG FBS (Sigma-Aldrich). IgG samples (purified from plasma, followed by 1:2 dilution) were added to the wells at a volume of 100 μL and incubated overnight at 4°C. Antigen-coated plates with IgG samples were washed and supplemented with 100 μL R10 (RPMI-1640 with glutamine +10% low IgG FBS), containing Monensin and Brefeldin A (BD Biosciences, 1:200) and PE-conjugated anti-CD107a antibody (1:1000). NK cells were isolated from a healthy donor as described above, added at a concentration of 2.5x10^6^ in a volume of 100 μL R10 per well, and incubated at 37°C and 5% CO_2_ for 5 h. Cells were transferred to a V-bottom plate and surface staining was performed for 15 min at 37°C, in 100 μL staining buffer containing 0.1 μL viability stain (Zombie NIR Fixable Viability Kit, Biolegend) and antibodies directed against human CD56 (1 μL), CD16 (2 μL), CD3, CD14 and CD19 (0.5 μL each). Subsequently, cells were fixed and permeabilized using eBioscience Intracellular Fixation & Permeabilization Buffers (Thermo Fisher Scientific), followed by intracellular staining with 100 μL Permeabilization Buffer containing antibodies against human IFNγ (1 μL) and MIP-1β (0.5 μL), for 15 min at 37°C (see key resources table for more details about antibodies). Flow cytometric analysis was performed with the Cytek Aurora (Cytek Biosciences). Doublets and dead cells were excluded from the analysis, and NK cells were identified as CD3^–^CD14^–^CD19^–^CD56^+^CD16^+/−^. As a measure of ADNKA capacity, frequencies of NK cells positive for degranulation marker CD107a and activation markers IFNγ and MIP-1β were determined (for gating strategy see Figure S3).

#### *N*-linked Fc glycan analysis

Fc *N*-glycosylation was analyzed after isolation of IgG from plasma samples using Protein A Dynabeads, followed by isolation of S1/S2-specific IgG and glycopeptide purification, adapted from Wieczorek et al.^90^ Briefly, 50 μL of plasma was incubated with 50 μL of Protein A Dynabeads suspension (Thermo Fisher Scientific) for 45 min. Afterward, the beads were washed with 2 × 50 μL of water and transferred into a new tube. The captured total IgG1, 2 and 4 were eluted with 2 × 50 μL of 100 mM formic acid. Ten microliters of the eluate were separated, dried in a vacuum centrifuge, and stored for further total IgG analysis. The remaining eluate was dried in a vacuum centrifuge and redissolved in 50 μL of water for isolation of S1- or S2-specific IgG. For this purpose, carboxylated microspheres were coated with the MERS-CoV S protein S1 and S2 subunits as described above, diluted to 5000 microspheres/μL, and 20 μL were added to the redissolved total IgG. After 45 min of incubation, the microspheres were washed and transferred into a new tube. The captured S1/S2-specific IgG1, 2 and 4 were eluted with 2 × 50 μL of 100 mM formic acid and dried in a vacuum centrifuge. Total and S1/S2-specific IgG were redissolved in 50 μL of 50 mM ammonium bicarbonate and tryptic digestion was performed by adding 2 μL of 0.5 μg/μL sequencing grade modified trypsin in resuspension buffer provided by the manufacturer (Promega). After overnight incubation at 37°C, the tryptic IgG peptides were dried in a vacuum centrifuge. For further purification of glycopeptides, a self-made cotton-HILIC micro-spin column was used.^91^ The conditioning was performed by using 3 × 50 μL of water and 3 × 50 μL of 80% acetonitrile in water (V/V). Dried IgG samples were redissolved in 50 μL of 80% acetonitrile in water (V/V) and applied to the column. The washing steps were as follows: 3 × 50 μL of 80% acetonitrile in water (V/V) and 3 × 50 μL of 80% acetonitrile in water containing 0.1% trifluoroacetic acid (V/V/V). The purified IgG glycopeptides were eluted with 6 × 50 μL of water, dried in a vacuum centrifuge, and stored at −20°C until measurement. For the Matrix-assisted laser desorption/ionization (MALDI) mass spectrometry analysis, the dried glycopeptides were dissolved in 4.5 μL of MS water. The sample (0.8 μL) was spotted onto a MALDI-Target and the same volume of saturated HCCA Matrix in 70% acetonitrile in water (V/V) was added. Once the droplet had dried, the measurement was performed on a RapifleX MALDI-TOF/TOF mass spectrometer (Bruker Daltonics). The calibration was carried out with the Peptide Calibration Standard II (Bruker Daltonics) and measurement was performed in reflectron negative ionization mode within a range of m/z 1000–5000. Raw spectra were processed with Flex Analysis (Bruker Daltonics), including baseline subtraction, smoothing, and peak picking. The abundance of each glycosylation trait was calculated by summing up the relative intensities of the subclass-specific glycopeptide signals belonging to a trait and normalizing it to the sum of all identified glycopeptide signal intensities, as described in detail by Schwedler et al.^92^ Baseline (V1D0) samples of each study participant were analyzed as reference controls to exclude background signals in the glycan analysis of S1- and S2-specific IgG1. These control samples didn’t reveal any signals corresponding to an m/z value of the analyzed Fc glycopeptides.

### Quantification and statistical analysis

Flow cytometry data was analyzed using the FlowJo software (v.10.7.2, Becton Dickinson, Franklin Lakes). Data analysis and visualization was performed using GraphPad Prism (v.9.5.1, Dotmatics, Boston, USA) and RStudio (2023.12.0 + 369 and R v. 4.3.2, The R Foundation, Vienna, Austria). The number of study participants (n), median, interquartile and min-max range for all experiments are shown in Tables S1–S3. Due to the small sample size and high number of immunological parameters assessed, no statistical significance testing was performed (see also limitations of the study). For the heatmap (Figure 1G) and circular barplots (Figure 2A), rescaling of the dataset was performed using the following formula: X = (X – Xmin)/(Xmax – Xmin). The correlations of V3D28 data (Figures 2B, 2C, and 3C–3E) were performed based on a non-parametric Spearman’s correlation.

### Additional resources

ClinicalTrials.gov Identifier: NCT03615911. https://clinicaltrials.gov/ct2/show/NCT03615911.

## Consortia

The members of the MVA-MERS-S-CEF study group are Etienne Bartels, Swantje Gundlach, Thomas Hesterkamp, Verena Krähling, Susan Lassen, My Linh Ly, Joseph H. Pötsch, Stefan Schmiedel, Asisa Volz, and Madeleine E. Zinser.

## References

[1] Conzade R., Grant R., Malik M.R., Elkholy A., Elhakim M., Samhouri D., Ben Embarek P.K., Van Kerkhove M.D. (2018). Reported Direct and Indirect Contact with Dromedary Camels among Laboratory-Confirmed MERS-CoV Cases. Viruses.

[2] Zaki A.M., van Boheemen S Fau - Bestebroer T.M., Bestebroer Tm Fau - Osterhaus A.D.M.E., Osterhaus Ad Fau - Fouchier R.A.M., Fouchier R.A. (2012). Isolation of a novel coronavirus from a man with pneumonia in Saudi Arabia. N. Engl. J. Med..

[3] Hui D.S., Azhar E.I., Kim Y.J., Memish Z.A., Oh M.D., Zumla A. (2018). Middle East respiratory syndrome coronavirus: risk factors and determinants of primary, household, and nosocomial transmission. Lancet Infect. Dis..

[4] WHO (2023). MERS Situation Update. https://applications.emro.who.int/docs/WHOEMCSR675E-eng.pdf.

[5] Memish Z.A., Perlman S., Van Kerkhove M.D., Zumla A. (2020). Middle East respiratory syndrome. Lancet.

[6] Yong C.Y., Ong H.K., Yeap S.K., Ho K.L., Tan W.S. (2019). Recent Advances in the Vaccine Development Against Middle East Respiratory Syndrome-Coronavirus. Front. Microbiol..

[7] Zumla A., Peiris M., Memish Z.A., Perlman S. (2024). Anticipating a MERS-like coronavirus as a potential pandemic threat. Lancet.

[8] Wang Q., Wong G., Lu G., Yan J., Gao G.F. (2016). MERS-CoV spike protein: Targets for vaccines and therapeutics. Antiviral Res..

[9] Hatmal M.m.M., Alshaer W., Al-Hatamleh M.A.I., Hatmal M., Smadi O., Taha M.O., Oweida A.J., Boer J.C., Mohamud R., Plebanski M. (2020). Comprehensive Structural and Molecular Comparison of Spike Proteins of SARS-CoV-2, SARS-CoV and MERS-CoV, and Their Interactions with ACE2. Cells.

[10] Modjarrad K., Roberts C.C., Mills K.T., Castellano A.R., Paolino K., Muthumani K., Reuschel E.L., Robb M.L., Racine T., Oh M.-d. (2019). Safety and immunogenicity of an anti-Middle East respiratory syndrome coronavirus DNA vaccine: a phase 1, open-label, single-arm, dose-escalation trial. Lancet Infect. Dis..

[11] Folegatti P.M., Bittaye M., Flaxman A., Lopez F.R., Bellamy D., Kupke A., Mair C., Makinson R., Sheridan J., Rohde C. (2020). Safety and immunogenicity of a candidate Middle East respiratory syndrome coronavirus viral-vectored vaccine: a dose-escalation, open-label, non-randomised, uncontrolled, phase 1 trial. Lancet Infect. Dis..

[12] Koch T., Dahlke C., Fathi A., Kupke A., Krähling V., Okba N.M.A., Halwe S., Rohde C., Eickmann M., Volz A. (2020). Safety and immunogenicity of a modified vaccinia virus Ankara vector vaccine candidate for Middle East respiratory syndrome: an open-label, phase 1 trial. Lancet Infect. Dis..

[13] Choi J.A., Kim J.O. (2022). Middle East Respiratory Syndrome coronavirus vaccine development: updating clinical studies using platform technologies. J. Microbiol..

[14] Song F., Fux R., Provacia L.B., Volz A., Eickmann M., Becker S., Osterhaus A.D., Haagmans B.L., Sutter G. (2013). Middle East respiratory syndrome coronavirus spike protein delivered by modified vaccinia virus Ankara efficiently induces virus-neutralizing antibodies. J. Virol..

[15] Fathi A., Dahlke C., Krähling V., Kupke A., Okba N.M.A., Raadsen M.P., Heidepriem J., Müller M.A., Paris G., Lassen S. (2022). Increased neutralization and IgG epitope identification after MVA-MERS-S booster vaccination against Middle East respiratory syndrome. Nat. Commun..

[16] Weskamm L.M., Fathi A., Raadsen M.P., Mykytyn A.Z., Koch T., Spohn M., Friedrich M., Haagmans B.L., Becker S., Sutter G. (2022). Persistence of MERS-CoV-spike-specific B cells and antibodies after late third immunization with the MVA-MERS-S vaccine. Cell Rep. Med..

[17] Plotkin S.A. (2008). Vaccines: correlates of vaccine-induced immunity. Clin. Infect. Dis..

[18] Siegrist C.-A., Plotkin S.A., Orenstein W.A., Offit P.A., Edwards K.M. (2018). Plotkin's Vaccines.

[19] Khoury D.S., Cromer D., Reynaldi A., Schlub T.E., Wheatley A.K., Juno J.A., Subbarao K., Kent S.J., Triccas J.A., Davenport M.P. (2021). Neutralizing antibody levels are highly predictive of immune protection from symptomatic SARS-CoV-2 infection. Nat. Med..

[20] Vanderven H.A., Kent S.J. (2020). The protective potential of Fc-mediated antibody functions against influenza virus and other viral pathogens. Immunol. Cell Biol..

[21] Lu L.L., Suscovich T.J., Fortune S.M., Alter G. (2018). Beyond binding: antibody effector functions in infectious diseases. Nat. Rev. Immunol..

[22] Tay M.Z., Wiehe K., Pollara J. (2019). Antibody-Dependent Cellular Phagocytosis in Antiviral Immune Responses. Front. Immunol..

[23] Vidarsson G., Dekkers G., Rispens T. (2014). IgG subclasses and allotypes: from structure to effector functions. Front. Immunol..

[24] Arnold K.B., Chung A.W. (2018). Prospects from systems serology research. Immunology.

[25] Zhang A., Stacey H.D., D’Agostino M.R., Tugg Y., Marzok A., Miller M.S. (2023). Beyond neutralization: Fc-dependent antibody effector functions in SARS-CoV-2 infection. Nat. Rev. Immunol..

[26] Raadsen M.P., Dahlke C., Fathi A., Lamers M.M., van den Doel P., Zaeck L.M., van Royen M.E., de Bruin E., Sikkema R., Koopmans M. (2023). Brief Report: Monkeypox Virus Cross-Neutralizing Antibodies in Clinical Trial Subjects Vaccinated with Modified Vaccinia Virus Ankara Encoding MERS-Coronavirus Spike Protein. J. Infect. Dis..

[27] Kreijtz J.H., Goeijenbier M., Moesker F.M., van den Dries L., Goeijenbier S., De Gruyter H.L., Lehmann M.H., Mutsert G., van de Vijver D.A., Volz A. (2014). Safety and immunogenicity of a modified-vaccinia-virus-Ankara-based influenza A H5N1 vaccine: a randomised, double-blind phase 1/2a clinical trial. Lancet Infect. Dis..

[28] Guardo A.C., Gómez C.E., Díaz-Brito V., Pich J., Arnaiz J.A., Perdiguero B., García-Arriaza J., González N., Sorzano C.O.S., Jiménez L. (2017). Safety and vaccine-induced HIV-1 immune responses in healthy volunteers following a late MVA-B boost 4 years after the last immunization. PLoS One.

[29] Barry H., Mutua G., Kibuuka H., Anywaine Z., Sirima S.B., Meda N., Anzala O., Eholie S., Bétard C., Richert L. (2021). Safety and immunogenicity of 2-dose heterologous Ad26.ZEBOV, MVA-BN-Filo Ebola vaccination in healthy and HIV-infected adults: A randomised, placebo-controlled Phase II clinical trial in Africa. PLoS Med..

[30] Tong X., McNamara R.P., Avendaño M.J., Serrano E.F., García-Salum T., Pardo-Roa C., Bertera H.L., Chicz T.M., Levican J., Poblete E. (2023). Waning and boosting of antibody Fc-effector functions upon SARS-CoV-2 vaccination. Nat. Commun..

[31] Shubin Z., Stanfield-Oakley S., Puangkaew J., Pitisutthithum P., Nitayaphan S., Gurunathan S., Sinangil F., Chariyalertsak S., Phanuphak N., Ake J.A. (2023). Additional boosting to the RV144 vaccine regimen increased Fc-mediated effector function magnitude but not durability. Aids.

[32] Cyster J.G., Allen C.D.C. (2019). B Cell Responses: Cell Interaction Dynamics and Decisions. Cell.

[33] Wishnie A.J., Chwat-Edelstein T., Attaway M., Vuong B.Q. (2021). BCR Affinity Influences T-B Interactions and B Cell Development in Secondary Lymphoid Organs. Front. Immunol..

[34] Sallusto F., Lanzavecchia A., Araki K., Ahmed R. (2010). From vaccines to memory and back. Immunity.

[35] Moriyama S., Adachi Y., Sato T., Tonouchi K., Sun L., Fukushi S., Yamada S., Kinoshita H., Nojima K., Kanno T. (2021). Temporal maturation of neutralizing antibodies in COVID-19 convalescent individuals improves potency and breadth to circulating SARS-CoV-2 variants. Immunity.

[36] Raadsen M., Dahlke C., Fathi A., Hardtke S., Klüver M., Krähling V., Gerresheim G.K., Mayer L., Mykytyn A.Z., Weskamm L.M. (2024). The Safety, Immunogenicity, and Optimal Dosing of an MVA-Based Vaccine Against MERS Coronavirus in Healthy Adults: A Phase 1b, Randomised, Placebo-Controlled, Double-Blind Clinical Trial. Lancet.

[37] Lu G., Hu Y., Wang Q., Qi J., Gao F., Li Y., Zhang Y., Zhang W., Yuan Y., Bao J. (2013). Molecular basis of binding between novel human coronavirus MERS-CoV and its receptor CD26. Nature.

[38] Xu J., Jia W., Wang P., Zhang S., Shi X., Wang X., Zhang L. (2019). Antibodies and vaccines against Middle East respiratory syndrome coronavirus. Emerg. Microbes Infect..

[39] Chen Y., Zhao X., Zhou H., Zhu H., Jiang S., Wang P. (2023). Broadly neutralizing antibodies to SARS-CoV-2 and other human coronaviruses. Nat. Rev. Immunol..

[40] Li C.J., Chang S.C. (2023). SARS-CoV-2 spike S2-specific neutralizing antibodies. Emerg. Microbes Infect..

[41] Du L., Yang Y., Zhou Y., Lu L., Li F., Jiang S. (2017). MERS-CoV spike protein: a key target for antivirals. Expert Opin. Ther. Targets.

[42] Silva R.P., Huang Y., Nguyen A.W., Hsieh C.L., Olaluwoye O.S., Kaoud T.S., Wilen R.E., Qerqez A.N., Park J.G., Khalil A.M. (2023). Identification of a conserved S2 epitope present on spike proteins from all highly pathogenic coronaviruses. Elife.

[43] Claireaux M., Caniels T.G., de Gast M., Han J., Guerra D., Kerster G., van Schaik B.D.C., Jongejan A., Schriek A.I., Grobben M. (2022). A public antibody class recognizes an S2 epitope exposed on open conformations of SARS-CoV-2 spike. Nat. Commun..

[44] Wang C., van Haperen R., Gutiérrez-Álvarez J., Li W., Okba N.M.A., Albulescu I., Widjaja I., van Dieren B., Fernandez-Delgado R., Sola I. (2021). A conserved immunogenic and vulnerable site on the coronavirus spike protein delineated by cross-reactive monoclonal antibodies. Nat. Commun..

[45] Shah P., Canziani G.A., Carter E.P., Chaiken I. (2021). The Case for S2: The Potential Benefits of the S2 Subunit of the SARS-CoV-2 Spike Protein as an Immunogen in Fighting the COVID-19 Pandemic. Front. Immunol..

[46] Zhou P., Song G., Liu H., Yuan M., He W.T., Beutler N., Zhu X., Tse L.V., Martinez D.R., Schäfer A. (2023). Broadly neutralizing anti-S2 antibodies protect against all three human betacoronaviruses that cause deadly disease. Immunity.

[47] Wang L., Shi W., Chappell James D., Joyce M.G., Zhang Y., Kanekiyo M., Becker Michelle M., van Doremalen N., Fischer R., Wang N. (2018). Importance of Neutralizing Monoclonal Antibodies Targeting Multiple Antigenic Sites on the Middle East Respiratory Syndrome Coronavirus Spike Glycoprotein To Avoid Neutralization Escape. J. Virol..

[48] Ng K.W., Faulkner N., Finsterbusch K., Wu M., Harvey R., Hussain S., Greco M., Liu Y., Kjaer S., Swanton C. (2022). SARS-CoV-2 S2-targeted vaccination elicits broadly neutralizing antibodies. Sci. Transl. Med..

[49] Tai W., Zhang X., Yang Y., Zhu J., Du L. (2022). Advances in mRNA and other vaccines against MERS-CoV. Transl. Res..

[50] Dolgin E. (2022). Pan-coronavirus vaccine pipeline takes form. Nat. Rev. Drug Discov..

[51] Zhao C., Xu J. (2018). Toward universal influenza virus vaccines: from natural infection to vaccination strategy. Curr. Opin. Immunol..

[52] Wang X., Sun L., Liu Z., Xing L., Zhu Y., Xu W., Xia S., Lu L., Jiang S. (2023). An engineered recombinant protein containing three structural domains in SARS-CoV-2 S2 protein has potential to act as a pan-human coronavirus entry inhibitor or vaccine antigen. Emerg. Microbes Infect..

[53] Impagliazzo A., Milder F., Kuipers H., Wagner M.V., Zhu X., Hoffman R.M., van Meersbergen R., Huizingh J., Wanningen P., Verspuij J. (2015). A stable trimeric influenza hemagglutinin stem as a broadly protective immunogen. Science.

[54] Nguyen-Contant P., Embong A.K., Kanagaiah P., Chaves Francisco A., Yang H., Branche Angela R., Topham David J., Sangster Mark Y. (2020). S Protein-Reactive IgG and Memory B Cell Production after Human SARS-CoV-2 Infection Includes Broad Reactivity to the S2 Subunit. mBio.

[55] Grobben M., van der Straten K., Brouwer P.J., Brinkkemper M., Maisonnasse P., Dereuddre-Bosquet N., Appelman B., Lavell A.A., van Vught L.A., Burger J.A. (2021). Cross-reactive antibodies after SARS-CoV-2 infection and vaccination. Elife.

[56] Camerini D., Randall A.Z., Trappl-Kimmons K., Oberai A., Hung C., Edgar J., Shandling A., Huynh V., Teng A.A., Hermanson G. (2021). Mapping SARS-CoV-2 Antibody Epitopes in COVID-19 Patients with a Multi-Coronavirus Protein Microarray. Microbiol. Spectr..

[57] Peng Y., Liu Y., Hu Y., Chang F., Wu Q., Yang J., Chen J., Teng S., Zhang J., He R. (2022). Monoclonal antibodies constructed from COVID-19 convalescent memory B cells exhibit potent binding activity to MERS-CoV spike S2 subunit and other human coronaviruses. Front. Immunol..

[58] Galipeau Y., Greig M., Liu G., Driedger M., Langlois M.A. (2020). Humoral Responses and Serological Assays in SARS-CoV-2 Infections. Front. Immunol..

[59] Murray S.M., Ansari A.M., Frater J., Klenerman P., Dunachie S., Barnes E., Ogbe A. (2023). The impact of pre-existing cross-reactive immunity on SARS-CoV-2 infection and vaccine responses. Nat. Rev. Immunol..

[60] Piccoli L., Park Y.J., Tortorici M.A., Czudnochowski N., Walls A.C., Beltramello M., Silacci-Fregni C., Pinto D., Rosen L.E., Bowen J.E. (2020). Mapping Neutralizing and Immunodominant Sites on the SARS-CoV-2 Spike Receptor-Binding Domain by Structure-Guided High-Resolution Serology. Cell.

[61] Abbott R.K., Crotty S. (2020). Factors in B cell competition and immunodominance. Immunol. Rev..

[62] Vatti A., Monsalve D.M., Pacheco Y., Chang C., Anaya J.M., Gershwin M.E. (2017). Original antigenic sin: A comprehensive review. J. Autoimmun..

[63] Rijkers G.T., van Overveld F.J. (2021). The “original antigenic sin” and its relevance for SARS-CoV-2 (COVID-19) vaccination. Clin. Immunol. Commun..

[64] Anderson E.M., Li S.H., Awofolaju M., Eilola T., Goodwin E., Bolton M.J., Gouma S., Manzoni T.B., Hicks P., Goel R.R. (2022). SARS-CoV-2 infections elicit higher levels of original antigenic sin antibodies compared with SARS-CoV-2 mRNA vaccinations. Cell Rep..

[65] Amanat F., Thapa M., Lei T., Ahmed S.M.S., Adelsberg D.C., Carreño J.M., Strohmeier S., Schmitz A.J., Zafar S., Zhou J.Q. (2021). SARS-CoV-2 mRNA vaccination induces functionally diverse antibodies to NTD, RBD, and S2. Cell.

[66] Irvine E.B., Alter G. (2020). Understanding the role of antibody glycosylation through the lens of severe viral and bacterial diseases. Glycobiology.

[67] Damelang T., Rogerson S.J., Kent S.J., Chung A.W. (2019). Role of IgG3 in Infectious Diseases. Trends Immunol..

[68] Shields R.L., Lai J., Keck R., O'Connell L.Y., Hong K., Meng Y.G., Weikert S.H.A., Presta L.G. (2002). Lack of fucose on human IgG1 N-linked oligosaccharide improves binding to human Fcgamma RIII and antibody-dependent cellular toxicity. J. Biol. Chem..

[69] Shinkawa T., Nakamura K., Yamane N., Shoji-Hosaka E., Kanda Y., Sakurada M., Uchida K., Anazawa H., Satoh M., Yamasaki M. (2003). The absence of fucose but not the presence of galactose or bisecting N-acetylglucosamine of human IgG1 complex-type oligosaccharides shows the critical role of enhancing antibody-dependent cellular cytotoxicity. J. Biol. Chem..

[70] Niwa R., Hatanaka S., Shoji-Hosaka E., Sakurada M., Kobayashi Y., Uehara A., Yokoi H., Nakamura K., Shitara K. (2004). Enhancement of the antibody-dependent cellular cytotoxicity of low-fucose IgG1 Is independent of FcgammaRIIIa functional polymorphism. Clin. Cancer Res..

[71] de Taeye S.W., Rispens T., Vidarsson G. (2019). The Ligands for Human IgG and Their Effector Functions. Antibodies.

[72] Mancardi D., Daëron M. (2014). Fc Receptors in Immune Responses. Reference Module in Biomedical Sciences.

[73] Van Coillie J., Pongracz T., Rahmöller J., Chen H.-J., Geyer C.E., van Vught L.A., Buhre J.S., Šuštić T., van Osch T.L.J., Steenhuis M. (2023). The BNT162b2 mRNA SARS-CoV-2 vaccine induces transient afucosylated IgG1 in naive but not in antigen-experienced vaccinees. EBioMedicine.

[74] Zou G., Ochiai H., Huang W., Yang Q., Li C., Wang L.X. (2011). Chemoenzymatic synthesis and Fcγ receptor binding of homogeneous glycoforms of antibody Fc domain. Presence of a bisecting sugar moiety enhances the affinity of Fc to FcγIIIa receptor. J. Am. Chem. Soc..

[75] Kapur R., Kustiawan I., Vestrheim A., Koeleman C.A., Visser R., Einarsdottir H.K., Porcelijn L., Jackson D., Kumpel B., Deelder A.M. (2014). A prominent lack of IgG1-Fc fucosylation of platelet alloantibodies in pregnancy. Blood.

[76] Pascal K.E., Coleman C.M., Mujica A.O., Kamat V., Badithe A., Fairhurst J., Hunt C., Strein J., Berrebi A., Sisk J.M. (2015). Pre- and postexposure efficacy of fully human antibodies against Spike protein in a novel humanized mouse model of MERS-CoV infection. Proc. Natl. Acad. Sci. USA.

[77] Houser K.V., Gretebeck L., Ying T., Wang Y., Vogel L., Lamirande E.W., Bock K.W., Moore I.N., Dimitrov D.S., Subbarao K. (2016). Prophylaxis With a Middle East Respiratory Syndrome Coronavirus (MERS-CoV)-Specific Human Monoclonal Antibody Protects Rabbits From MERS-CoV Infection. J. Infect. Dis..

[78] Corti D., Zhao J., Pedotti M., Simonelli L., Agnihothram S., Fett C., Fernandez-Rodriguez B., Foglierini M., Agatic G., Vanzetta F. (2015). Prophylactic and postexposure efficacy of a potent human monoclonal antibody against MERS coronavirus. Proc. Natl. Acad. Sci. USA.

[79] Johnson R.F., Bagci U., Keith L., Tang X., Mollura D.J., Zeitlin L., Qin J., Huzella L., Bartos C.J., Bohorova N. (2016). 3B11-N, a monoclonal antibody against MERS-CoV, reduces lung pathology in rhesus monkeys following intratracheal inoculation of MERS-CoV Jordan-n3/2012. Virology.

[80] Ullah I., Prévost J., Ladinsky M.S., Stone H., Lu M., Anand S.P., Beaudoin-Bussières G., Symmes K., Benlarbi M., Ding S. (2021). Live imaging of SARS-CoV-2 infection in mice reveals that neutralizing antibodies require Fc function for optimal efficacy. Immunity.

[81] Schäfer A., Muecksch F., Lorenzi J.C.C., Leist S.R., Cipolla M., Bournazos S., Schmidt F., Maison R.M., Gazumyan A., Martinez D.R. (2021). Antibody potency, effector function, and combinations in protection and therapy for SARS-CoV-2 infection in vivo. J. Exp. Med..

[82] Shiakolas A.R., Kramer K.J., Wrapp D., Richardson S.I., Schäfer A., Wall S., Wang N., Janowska K., Pilewski K.A., Venkat R. (2021). Cross-reactive coronavirus antibodies with diverse epitope specificities and Fc effector functions. Cell Rep. Med..

[83] Mackin S.R., Desai P., Whitener B.M., Karl C.E., Liu M., Baric R.S., Edwards D.K., Chicz T.M., McNamara R.P., Alter G., Diamond M.S. (2023). Fc-γR-dependent antibody effector functions are required for vaccine-mediated protection against antigen-shifted variants of SARS-CoV-2. Nat. Microbiol..

[84] Goldblatt D., Alter G., Crotty S., Plotkin S.A. (2022). Correlates of protection against SARS-CoV-2 infection and COVID-19 disease. Immunol. Rev..

[85] Bartsch Y.C., Tong X., Kang J., Avendaño M.J., Serrano E.F., García-Salum T., Pardo-Roa C., Riquelme A., Cai Y., Renzi I. (2022). Omicron variant Spike-specific antibody binding and Fc activity are preserved in recipients of mRNA or inactivated COVID-19 vaccines. Sci. Transl. Med..

[86] Zohar T., Alter G. (2020). Dissecting antibody-mediated protection against SARS-CoV-2. Nat. Rev. Immunol..

[87] Mykytyn A.Z., Breugem T.I., Riesebosch S., Schipper D., van den Doel P.B., Rottier R.J., Lamers M.M., Haagmans B.L. (2021). SARS-CoV-2 entry into human airway organoids is serine protease-mediated and facilitated by the multibasic cleavage site. Elife.

[88] Watanabe Y., Aoki-Kinoshita K.F., Ishihama Y., Okuda S. (2021). GlycoPOST realizes FAIR principles for glycomics mass spectrometry data. Nucleic Acids Res..

[89] Brown E.P., Licht A.F., Dugast A.S., Choi I., Bailey-Kellogg C., Alter G., Ackerman M.E. (2012). High-throughput, multiplexed IgG subclassing of antigen-specific antibodies from clinical samples. J. Immunol. Methods.

[90] Wieczorek M., Braicu E.I., Oliveira-Ferrer L., Sehouli J., Blanchard V. (2020). Immunoglobulin G Subclass-Specific Glycosylation Changes in Primary Epithelial Ovarian Cancer. Front. Immunol..

[91] Selman M.H., Hemayatkar M., Deelder A.M., Wuhrer M. (2011). Cotton HILIC SPE microtips for microscale purification and enrichment of glycans and glycopeptides. Anal. Chem..

[92] Schwedler C., Grzeski M., Kappert K., Rust J., Heymann G., Hoppe B., Blanchard V. (2022). Coronavirus Disease 2019-Related Alterations of Total and Anti-Spike IgG Glycosylation in Relation to Age and Anti-Spike IgG Titer. Front. Microbiol..

