# Supplementary material for: Dissecting humoral immune responses to an MVA-vectored MERS-CoV vaccine in humans using a systems serology approach
Source: iScience. 2024 Jul 8;27(8):110470. doi: 10.1016/j.isci.2024.110470 (PMC11325358; doi:10.1016/j.isci.2024.110470)
Supplement: Document S1. Figures S1–S4 and Tables S1–S5 [file mmc1.pdf]

iScience, Volume *27*

Supplemental information

Dissecting humoral immune responses to an MVA-vectored MERS-CoV vaccine in humans using a systems serology approach

Leonie M. Weskamm, Paulina Tarnow, Charlotte Harms, Melanie Huchon, Matthijs P. Raadsen, Monika Friedrich, Laura Rübenacker, Cordula Grüttner, Mariana G. Garcia, the MVA-MERS-S-CEF study group, Till Koch, Stephan Becker, Gerd Sutter, Edouard Lhomme, Bart L. Haagmans, Anahita Fathi, Sandra M. Blois, Christine Dahlke, Laura Richert, and Marylyn M. Addo

Supplemental Figures

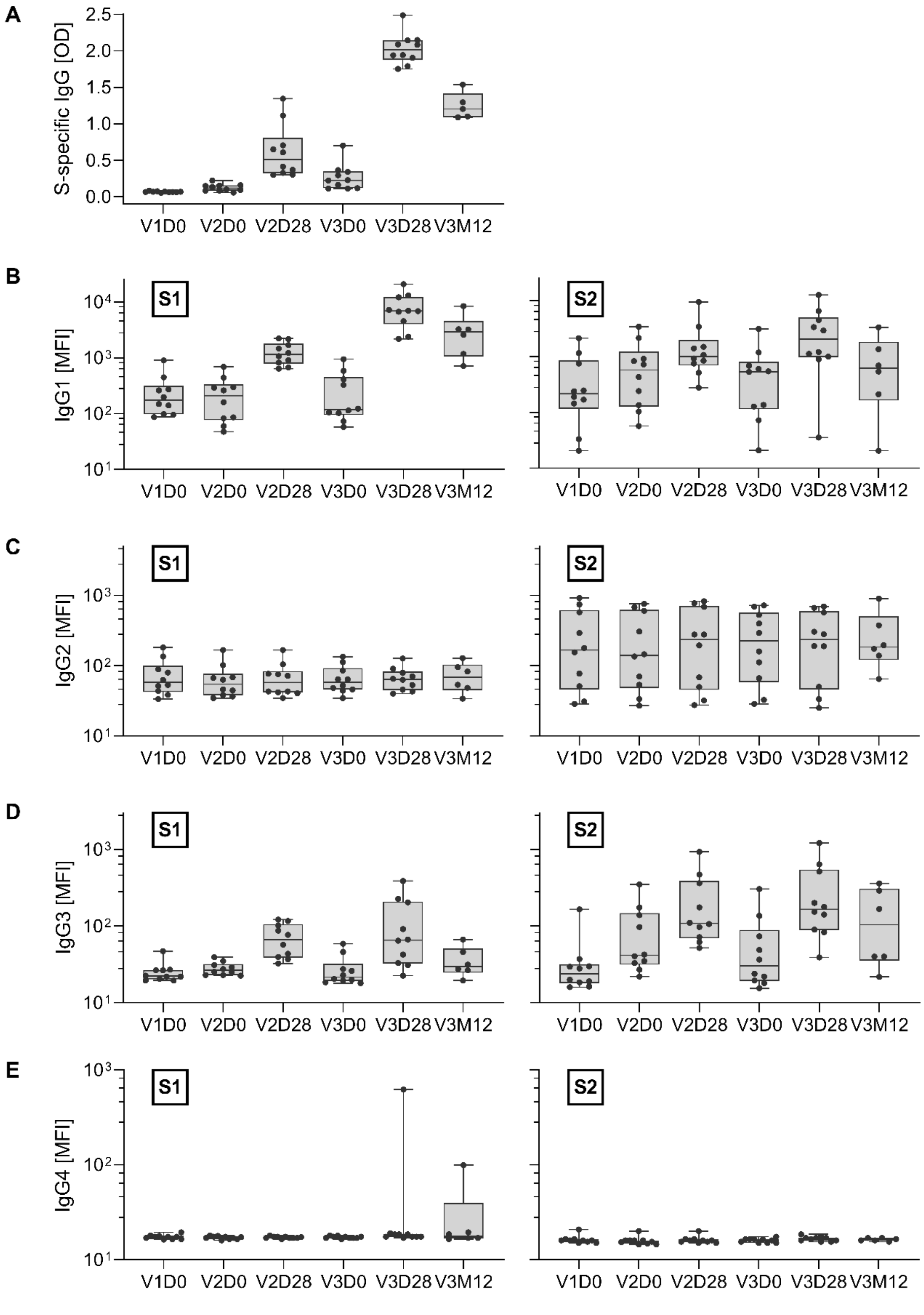

Figure S1. Longitudinal dynamics of MERS-CoV spike (S)-specific antibody titers, related to Figure 1B-C. A) S-specific IgG as measured by ELISA. **B-E)** IgG1-4 subclasses specific to S1- (left) and S2- (right) S protein subunits, measured by multiplex bead-based immunoassay. Boxplots indicate median, IQR and min-max range. Dots represent individual values. Descriptive statistics are reported in Table S1. OD: optical density; MFI: median fluorescence intensity.

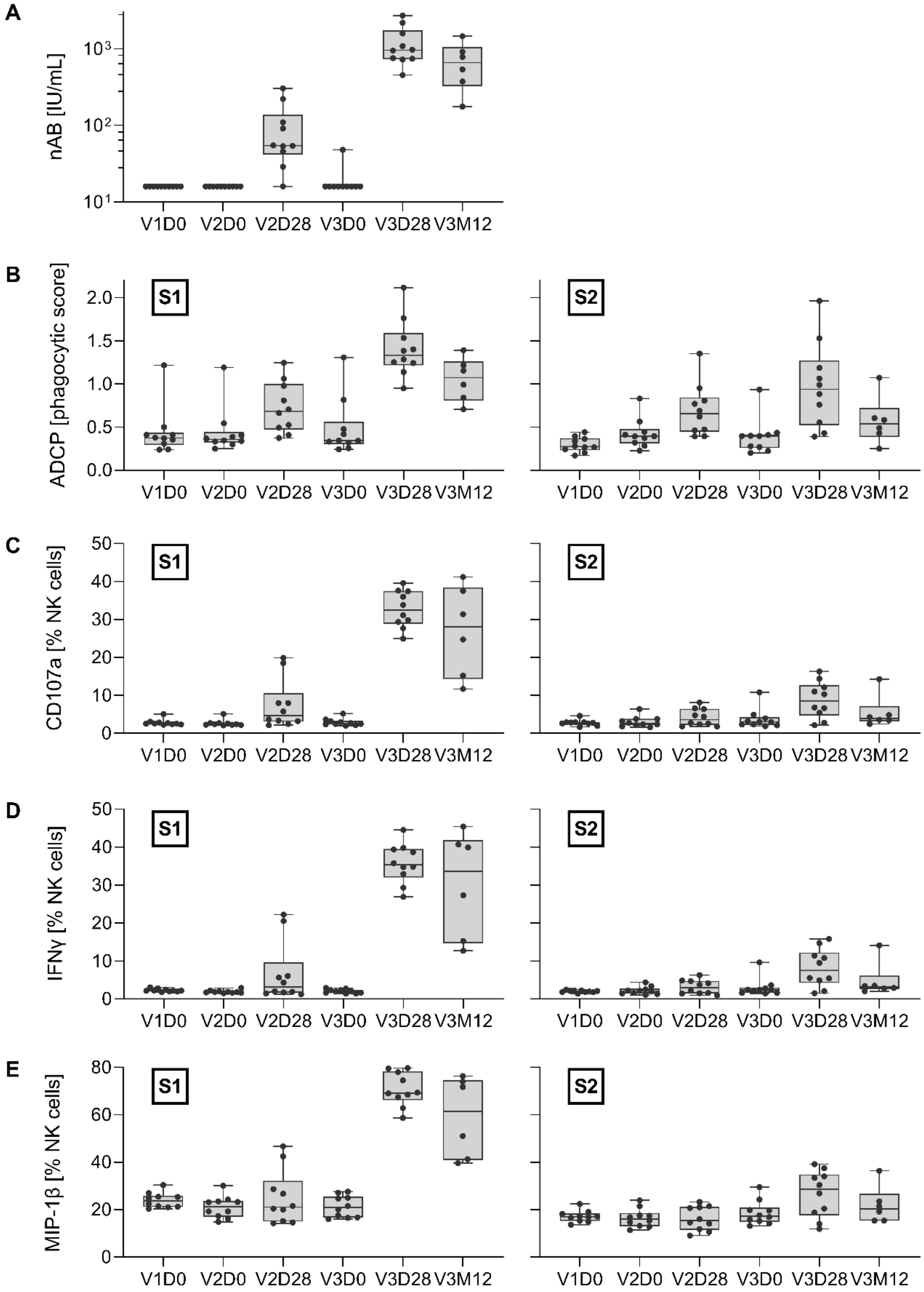

Figure S2. Longitudinal dynamics of MERS-CoV spike (S)-specific antibody functionality, related to Figure 1D-F. A) neutralizing antibodies (nAB) as measured by pseudovirus neutralization assay. **B-E)** Fc-mediated antibody functions specific to S1- (left) and S2- (right) S protein subunits, as measured by functional assays with flow cytometric readouts. **B)** Antibody-dependent cellular phagocytosis (ADCP). **C-E)** Antibody-dependent NK cell activation (ADNKA), represented by activation markers CD107a, IFNγ and MIP-1β. Boxplots indicate median, IQR and min-max range. Dots represent individual values. Descriptive statistics are reported in Table S2.

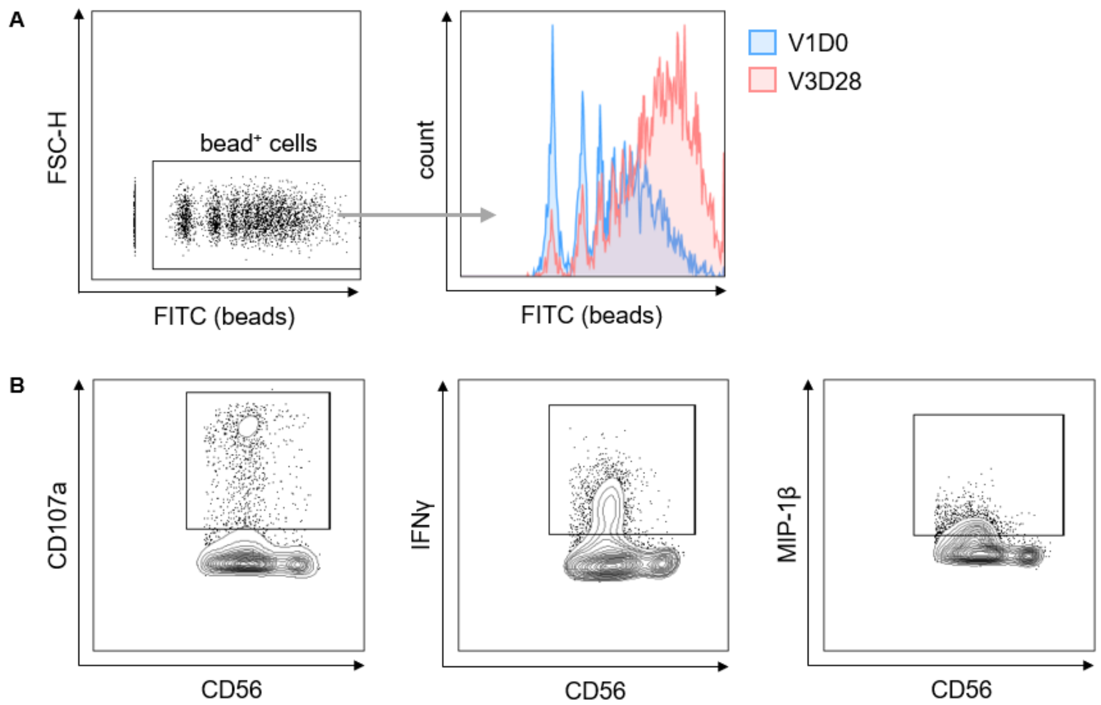

Figure S3. Gating strategies for flow cytometric readout of functional antibody assays, related to Figure 1E-F and STAR methods. A) Antibody-dependent cellular phagocytosis (ADCP) assay. After exclusion of doublets and dead cells, THP-1 cells with internalized beads were identified based on their FITC signal (left panel). Exemplary histograms of the FITC signal of bead-positive THP-1 cells are superimposed for time points V1D0 and V3D28 (right panel). The phagocytic score was calculated based on the percentage and mean fluorescence intensity (MFI) of FITC-positive THP-1 cells [% ($FITC^+$ cells) x MFI ($FITC^+$ cells)/1000]. **B)** Antibody-dependent NK cell activation (ADNKA) assay. After exclusion of doublets and dead cells, NK cells were identified based on their expression of CD56. ADNKA was assessed by expression of CD107a, IFNγ and MIP-1β, as shown in representative contour plots for time point V3D28.

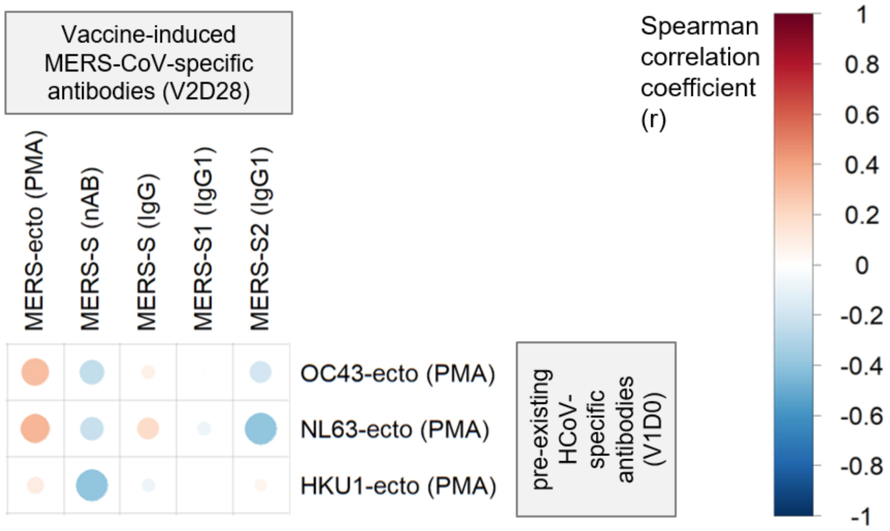

Figure S4. Spearman correlation analysis between pre-existing HCoV-specific antibody levels and vaccine-induced MERS-CoV-specific antibody levels, related to Figure 1. MERS-CoV spike-specific antibodies at V2D28 were measured by protein microarray (PMA), pseudovirus neutralization assay (nAB), ELISA (IgG), and bead-based multiplex immunoassay (IgG1). Antibodies against common cold HCoVs (HCoV-OC43, HCoV-NL63, HCoV-HKU1) were measured by PMA at baseline prior to vaccination (V1D0). Color and size of circles indicate the Spearman correlation coefficient (r). S: spike, ecto: spike ectodomain, S1: spike S1 subunit, S2: spike S2 subunit.

Supplemental Tables

Table S1. Descriptive statistics of IgG and subclass levels at longitudinal time points, related to Figure 1B-C.

		V1D0	V2D0	V2D28	V3D0	V3D28	V3M12
**S-specific IgG [OD]**	Sample size	10	10	10	10	10	5
	Minimum	0.06	0.06	0.30	0.11	1.75	1.09
	25% Percentile	0.06	0.08	0.32	0.12	1.88	1.10
	Median	0.07	0.12	0.51	0.23	2.01	1.21
	75% Percentile	0.07	0.16	0.81	0.35	2.15	1.42
	Maximum	0.09	0.22	1.35	0.70	2.49	1.54
**S1-specific IgG1 [MFI]**	Sample size	10	10	10	10	10	6
	Minimum	87	48	641	58	2185	714
	25% Percentile	98	77	783	95	3982	1053
	Median	173	211	1144	118	6898	2890
	75% Percentile	316	333	1799	454	12337	4538
	Maximum	897	688	2218	947	20678	8402
**S2-specific IgG1 [MFI]**	Sample size	10	10	10	10	10	6
	Minimum	21	57	277	21	36	21
	25% Percentile	116	126	687	114	968	163
	Median	214	574	991	532	2053	612
	75% Percentile	848	1214	1973	805	4998	1836
	Maximum	2122	3402	9419	3120	12616	3321
**S1-specific IgG2 [MFI]**	Sample size	10	10	10	10	10	6
	Minimum	34	35	35	35	40	34
	25% Percentile	42	38	42	46	45	45
	Median	58	55	58	58	64	68
	75% Percentile	100	77	84	92	82	104
	Maximum	182	167	167	134	127	128
**S2-specific IgG2 [MFI]**	Sample size	10	10	10	10	10	6
	Minimum	29	27	28	29	25	65
	25% Percentile	46	49	45	58	46	121
	Median	167	140	235	226	235	184
	75% Percentile	617	621	710	570	595	507
	Maximum	915	758	827	717	696	899
**S1-specific IgG3 [MFI]**	Sample size	10	10	10	10	10	6
	Minimum	20	23	33	18	23	20
	25% Percentile	20	23	39	19	33	25
	Median	22	27	67	22	66	30
	75% Percentile	27	32	106	32	210	51
	Maximum	47	39	122	59	389	67
**S2-specific IgG3 [MFI]**	Sample size	10	10	10	10	10	6
	Minimum	16	22	52	16	39	22
	25% Percentile	18	32	70	19	88	35
	Median	24	42	109	30	166	104
	75% Percentile	32	148	391	89	546	308
	Maximum	165	351	934	304	1217	361
**S1-specific IgG4 [MFI]**	Sample size	10	10	10	10	10	6
	Minimum	16	16	17	17	17	17
	25% Percentile	17	17	17	17	18	17
	Median	18	17	17	17	18	18
	75% Percentile	18	18	18	18	19	40
	Maximum	20	18	18	18	621	100
**S2-specific IgG4 [MFI]**	Sample size	10	10	10	10	10	6
	Minimum	15	15	15	15	16	16
	25% Percentile	15	15	15	15	16	16
	Median	16	16	16	16	17	16
	75% Percentile	17	16	17	17	17	17
	Maximum	21	20	20	18	19	17

Table S2. Descriptive statistics of neutralizing and Fc-mediated antibody functionality at longitudinal time points, related to Figure 1D-F.

		V1D0	V2D0	V2D28	V3D0	V3D28	V3M12
**nAB [IU/mL]**	Sample size	10	10	10	10	10	6
	Minimum	16	16	16	16	454.5	176.1
	25% Percentile	16	16	41.4	16	736.3	325.1
	Median	16	16	54.5	16	960.9	659.7
	75% Percentile	16	16	137.6	16	1734	1052
	Maximum	16	16	304.9	47.9	2714	1457
**S1-specific ADCP [phagocytic score]**	Sample size	10	10	10	10	10	6
	Minimum	0.2	0.3	0.4	0.2	0.9	0.7
	25% Percentile	0.3	0.3	0.5	0.3	1.2	0.8
	Median	0.4	0.4	0.7	0.3	1.3	1.1
	75% Percentile	0.4	0.4	1.0	0.6	1.6	1.3
	Maximum	1.2	1.2	1.2	1.3	2.1	1.4
**S2-specific ADCP [phagocytic score]**	Sample size	10	10	10	10	10	6
	Minimum	0.2	0.2	0.4	0.2	0.4	0.3
	25% Percentile	0.2	0.3	0.4	0.3	0.5	0.4
	Median	0.3	0.4	0.7	0.4	0.9	0.5
	75% Percentile	0.4	0.5	0.8	0.4	1.3	0.7
	Maximum	0.4	0.8	1.4	0.9	2.0	1.1
**S1-specific ADNKA [% of $CD107a^+$ NK cells]**	Sample size	10	10	10	10	10	6
	Minimum	2.3	2.1	2.2	2.0	24.9	11.7
	25% Percentile	2.4	2.3	3.0	2.3	28.9	14.3
	Median	2.6	2.4	4.7	2.6	32.5	28.1
	75% Percentile	2.9	2.7	10.6	3.3	37.5	38.4
	Maximum	5.1	5.1	19.9	5.2	39.6	41.2
**S2-specific ADNKA [% of $CD107a^+$ NK cells]**	Sample size	10	10	10	10	10	6
	Minimum	1.7	1.6	1.8	1.9	2.2	2.5
	25% Percentile	2.4	2.1	2.3	2.3	4.7	3.2
	Median	2.8	2.6	3.6	3.0	8.5	3.9
	75% Percentile	2.9	3.8	6.4	4.2	12.7	7.2
	Maximum	4.6	6.4	8.1	10.8	16.3	14.3
**S1-specific ADNKA [% of $IFN\gamma^+$ NK cells]**	Sample size	10	10	10	10	10	6
	Minimum	1.8	1.5	1.2	1.4	26.9	12.7
	25% Percentile	2.0	1.6	1.7	1.6	32.0	14.7
	Median	2.2	1.8	3.2	2.0	35.3	33.6
	75% Percentile	2.5	2.1	9.7	2.4	39.5	41.9
	Maximum	3.1	2.9	22.2	2.9	44.5	45.4
**S2-specific ADNKA [% of $IFN\gamma^+$ NK cells]**	Sample size	10	10	10	10	10	6
	Minimum	1.4	1.1	1.0	1.5	1.5	2.0
	25% Percentile	1.8	1.4	1.5	1.6	4.2	2.6
	Median	2.0	2.1	3.0	2.4	7.5	3.1
	75% Percentile	2.2	2.7	4.7	3.0	12.2	6.1
	Maximum	2.5	4.4	6.3	9.6	15.8	14.1
**S1-specific ADNKA [% of MIP-$1\beta^+$ NK cells]**	Sample size	10	10	10	10	10	6
	Minimum	20.4	14.9	14.3	16.1	58.7	39.6
	25% Percentile	21.2	17.0	15.1	16.7	66.3	40.9
	Median	23.7	21.3	21.1	20.9	69.2	61.4
	75% Percentile	25.9	23.7	32.2	25.6	78.4	74.6
	Maximum	30.4	30.1	46.7	27.6	79.7	76.3
**S2-specific ADNKA [% of MIP-$1\beta^+$ NK cells]**	Sample size	10	10	10	10	10	6
	Minimum	13.7	11.4	9.1	13.2	11.9	15.4
	25% Percentile	15.3	13.1	11.5	15.0	17.6	15.4
	Median	17.1	16.1	15.4	17.3	28.7	20.4
	75% Percentile	18.5	18.6	21.2	21.0	34.9	26.7
	Maximum	22.4	23.9	23.3	29.5	39.2	36.4

Table S3. IgG1 *N*-linked glycan species as detected by mass spectrometry, related to Figure 3.

Glycan composition	Glycan Structure	***m/z*** **[M-H]⁻ of detected glycoforms (Da)**
G0F	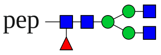	2632.04
G1F	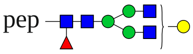	2794.09
G2F	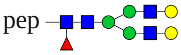	2956.14
G0FN	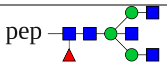	2835.12
G1FN	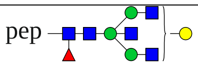	2997.17
G2FN	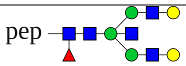	3159.22
G1FS1	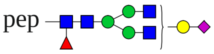	3085.18
G2FS1	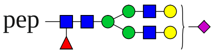	3247.24
Mono G0F	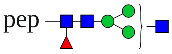	2428.96
Mono G1F	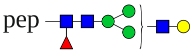	2591.01
G0	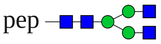	2485.98
G1	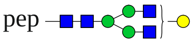	2648.03
G2	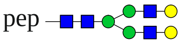	2810.08
G0N	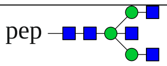	2689.06
G1N	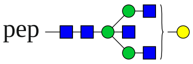	2851.11
G2N	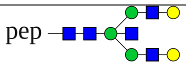	3013.16
G1S1	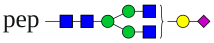	2939.14
G2S1	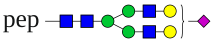	3101.18
Glycan compositions are indicted as G (galactose), F (fucose), N (bisecting N-acetylglucosamine), S (sialic acid), Mono (monoantennary). Schematic representations indicate N-acetylglucosamine (blue square), fucose (red triangle), mannose (green circle), galactose (yellow circle) and sialic acid (pink diamond), linked to a peptide moiety (pep).		

Table S4. Descriptive statistics of individual IgG1 *N*-linked Fc glycan species (relative abundance) at time point V3D28, related to Figure 3.

		S1-specific	S2-specific	total
**G0F [%]**	Sample size	10	10	10
	Minimum	14.3	10.3	13.7
	25% Percentile	17.3	17.2	15.7
	Median	20.9	19.9	21.9
	75% Percentile	22.5	23.4	23.6
	Maximum	25.7	26.9	26.8
**G1F [%]**	Sample size	10	10	10
	Minimum	28.8	25	25.8
	25% Percentile	30.3	30.2	29.1
	Median	33.1	32.8	31.1
	75% Percentile	36.7	36.7	34.2
	Maximum	47.9	40.4	38.9
**G2F [%]**	Sample size	10	10	10
	Minimum	17.1	12.3	13.2
	25% Percentile	19.3	17.3	14.1
	Median	21.8	20.6	15.8
	75% Percentile	29.6	24.8	19.1
	Maximum	34.6	31	28.7
**G0FN [%]**	Sample size	10	10	10
	Minimum	0.0	0.0	1.2
	25% Percentile	0.0	1.9	2.2
	Median	0.0	2.7	2.7
	75% Percentile	2.6	3.9	3.9
	Maximum	3.5	6.0	4.5
**G1FN [%]**	Sample size	10	10	10
	Minimum	0.0	4.2	2.7
	25% Percentile	0.0	4.8	4.5
	Median	4.9	5.2	5.2
	75% Percentile	5.6	5.6	5.7
	Maximum	6.5	6.0	6.8
**G2FN [%]**	Sample size	10	10	10
	Minimum	0.0	0.0	0.5
	25% Percentile	0.0	0.0	0.7
	Median	0.0	0.0	1.1
	75% Percentile	1.1	0.3	1.3
	Maximum	1.2	1.9	2.8
**G1FS1 [%]**	Sample size	10	10	10
	Minimum	0.0	0.0	0.2
	25% Percentile	0.0	0.0	0.4
	Median	0.0	0.0	0.5
	75% Percentile	0.0	0.0	0.6
	Maximum	0.0	0.0	2.5
**G2FS1 [%]**	Sample size	10	10	10
	Minimum	0.0	0.0	1.2
	25% Percentile	1.6	1.7	1.9
	Median	4.1	3.8	2.7
	75% Percentile	7.2	4.1	3.2
	Maximum	8.0	4.1	3.7
**Mono G0F [%]**	Sample size	10	10	10
	Minimum	0.0	0.0	2.2
	25% Percentile	0.0	0.0	2.6
	Median	3.5	3.1	2.9
	75% Percentile	5.1	3.2	3.0
	Maximum	6.2	3.7	4.1
**Mono G1F [%]**	Sample size	10	10	10
	Minimum	0.0	0.0	0.5
	25% Percentile	0.0	0.0	1.0
	Median	0.0	0.0	1.6
	75% Percentile	2.3	0.5	1.9
	Maximum	5.7	2.0	3.4

G0 [%]	Sample size	10	10	10
	Minimum	0.0	0.0	1.2
	25% Percentile	0.0	0.0	1.8
	Median	0.0	1.5	2.7
	75% Percentile	2.4	2.5	3.9
	Maximum	4.3	5.3	4.5
G1 [%]	Sample size	10	10	10
	Minimum	0.0	4.1	4.1
	25% Percentile	0.0	4.5	4.4
	Median	4.1	5.1	5.1
	75% Percentile	5.4	7.7	6.3
	Maximum	8.9	9.6	9.6
G2 [%]	Sample size	10	10	10
	Minimum	0.0	0.0	2.3
	25% Percentile	0.0	2.8	2.4
	Median	2.8	3.5	3.3
	75% Percentile	4.8	5.5	4.2
	Maximum	8.0	6.7	6.2
G0N [%]	Sample size	10	10	10
	Minimum	0.0	0.0	0.0
	25% Percentile	0.0	0.0	0.0
	Median	0.0	0.0	0.0
	75% Percentile	0.0	0.0	0.5
	Maximum	0.0	0.0	0.6
G1N [%]	Sample size	10	10	10
	Minimum	0.0	0.0	0.0
	25% Percentile	0.0	0.0	0.5
	Median	0.0	0.0	0.7
	75% Percentile	0.0	0.0	1.3
	Maximum	0.8	1.2	2.7
G2N [%]	Sample size	10	10	10
	Minimum	0.0	0.0	0.0
	25% Percentile	0.0	0.0	0.0
	Median	0.0	0.0	0.0
	75% Percentile	0.0	0.0	0.1
	Maximum	0.0	0.0	0.2
G1S1 [%]	Sample size	10	10	10
	Minimum	0.0	0.0	0.0
	25% Percentile	0.0	0.0	0.0
	Median	0.0	0.8	0.0
	75% Percentile	0.0	2.0	0.0
	Maximum	2.1	2.4	0.9
G2S1 [%]	Sample size	10	10	10
	Minimum	0.0	0.0	0.3
	25% Percentile	0.0	0.0	0.4
	Median	0.0	0.0	0.6
	75% Percentile	0.0	0.0	0.7
	Maximum	0.0	1.0	2.4

Table S5. Descriptive statistics of major glycosylation traits of IgG1 *N*-linked Fc glycans (relative abundance) at time point V3D28, related to Figure 3.

		S1-specific	S2-specific	total
**Fucosylation [%]**	Sample size	10	10	10
	Minimum	81.3	79.7	79.2
	25% Percentile	89.4	82.6	81.4
	Median	92.0	88.5	88.7
	75% Percentile	97.2	91.2	89.4
	Maximum	100.0	93.4	89.6
**Galactosylation [%]**	Sample size	10	10	10
	Minimum	46.0	41.4	41.6
	25% Percentile	48.1	46.2	44.0
	Median	50.3	49.0	47.4
	75% Percentile	57.4	55.7	50.9
	Maximum	67.4	61.6	59.1
**Bisection [%]**	Sample size	10	10	10
	Minimum	0.0	5.4	4.5
	25% Percentile	2.0	7.4	8.4
	Median	5.8	8.4	9.1
	75% Percentile	7.6	9.8	11.5
	Maximum	10.6	10.1	12.9
**Sialylation [%]**	Sample size	10	10	10
	Minimum	0.0	0.0	1.7
	25% Percentile	1.6	3.5	3.05
	Median	4.7	4.1	3.7
	75% Percentile	7.2	5.9	4.3
	Maximum	8.0	6.1	8.6
